# CO_2_ adsorption performance of template free zeolite A and X synthesized from rice husk ash as silicon source†

**DOI:** 10.1039/d2ra04052b

**Published:** 2022-08-17

**Authors:** Jayaprakash Madhu, Agilan Santhanam, Muthukumarasamy Natarajan, Dhayalan Velauthapillai

**Affiliations:** Department of Physics, Coimbatore Institute of Technology Coimbatore-641014 Tamil Nadu India; Faculty of Engineering and Science, Western Norway University of Applied Sciences 5063 Bergen Norway Dhayalan.Velauthapillai@hvl.no

## Abstract

In this work, zeolite NaA (RA) and NaX (RX) have been successfully synthesized using rice husk ash and it is a low cost synthesis process and it does not produce environmental hazards. Sodium silicate (SS) is extracted from rice husk ash which is an alternative silica source for zeolite synthesis. The zeolites are prepared by using a SS silica source extracted from the rice husk ash, and it has been used as an adsorbent for the CO_2_ adsorption process which may help in controlling the global warming problems. The zeolites are synthesized by a hydrothermal method without using any organic templating agent. FESEM and TEM micrographs revealed that the synthesized zeolites RA and RX have “Ice cube” and octahedral morphology respectively. From the N_2_ sorption studies, the BET surface area of the synthesized zeolites have been found and are 106.25 m^2^ g^−1^ and 512.79 m^2^ g^−1^ respectively. The maximum CO_2_ adsorption capacities of zeolite RA and RX are 2.22 and 2.45 mmol g^−1^, respectively at a temperature of 297.15 K. The recorded data are fitted by using non-linear adsorption isotherm models of Langmuir, Freundlich and Toth isotherm models. The fitted isotherm models are observed to be a type I adsorption isotherm according to the IUPAC classification criterion.

## Abstract 2

In this work, zeolite NaA (RA) and NaX (RX) have been successfully synthesized using rice husk ash as source and it is a low cost synthesis process and it does not produce any environmental hazards.

## Graphical abstract

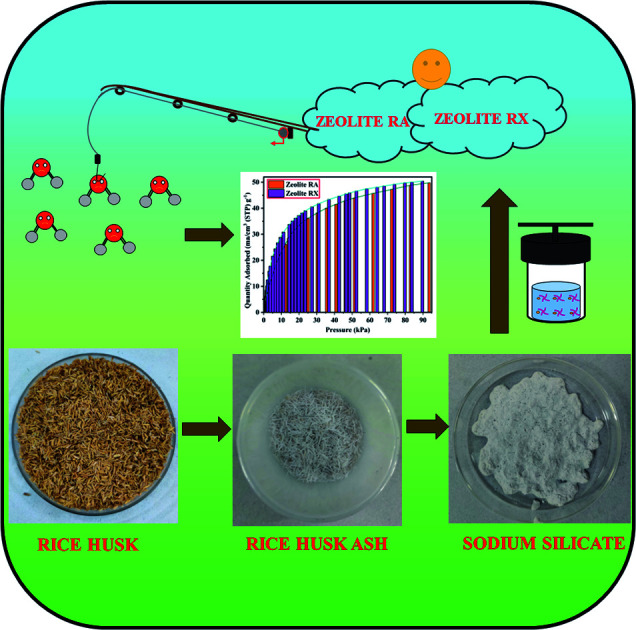

## 1. Introduction

In recent years, climate change and global warming arising due to the rapid rise of anthropogenic CO_2_ emission are considered as a major threat to the environment. The emission of CO_2_ has risen significantly from 280 ppm to 410 ppm in the span of 1760–2020 and has resulted in global warming and other serious concerns leading to environmental problems.^1^ During the last 5 years, the amount of CO_2_ emission has surpassed the maximum limit of 100 ppm and by the year of 2050, it is projected to exceed 500 ppm.^2^ Though the availability of alternative renewable and nonconventional energy resources have been identified, fossil fuels remain the world's primarily used energy resource and finding replacements for them is a big challenge.^3,4^ The excessive consumption of fossil fuels in the sectors of power generation, industry and transportation has led to the maximum amount of CO_2_ emission into the atmosphere. Due to this, the Intergovernmental Panel on Climate Change (IPCC) has emphasized that there is an urgent need for CO_2_ reduction and the implementation of necessary laws and regulations.^4^ In this context, the International Energy Agency (IEA) has highlighted, in the report of Energy Perspective 2020, that carbon capture and storage will play a key for the success of the Paris agreement.^5,6^ The average global temperature has climbed by 1 °C due to the excessive release of greenhouse emissions. To avoid dangerous climate change, serious measures are stated in the Paris Agreement to reduce the global temperature by 2 °C.^7^ In the year 2020, as a result of COVID-19 forced solitary confinement, the global CO_2_ emission has decreased by 5% compared to 2019. This decrease in CO_2_ emission has been achieved because of 8% reduction in the usage of coal, 2.3% reduction in usage of natural gas and 4.5% reduction in the usage of oil respectively. Hence, these results clearly indicate that fossil fuels play the lead role in global CO_2_ emission.^7,8^

As of late serious measures are adopted to reduce CO_2_ emission and thereby reduce global warming. At present, the methods used for CO_2_ capture and separation use solid adsorbents because they exhibit good adsorption capacity and shape selectivity.^9^ The capable solid adsorbents for CO_2_ capture must be available at low cost, should exhibit selective adsorption of CO_2_ rather than other gases, should have high surface area and large pore volume with the feasibility of rapid intraparticle diffusion. In addition to that, they should possess good cyclic stability along with high mechanical and chemical strength and should follow a simple regeneration process for repetitive adsorption.^9–11^ For the gas separation process, many solid porous adsorbents have been widely used. Solid adsorbents of different types having metal–organic framework (MOF) are well known for their variable pore surface and high surface area and they act as good CO_2_ adsorbent and they also have a better selectivity for adsorption.^12^ At lower temperatures, the MOF exhibits better CO_2_ adsorption, due to the presence of unsaturated metal ions in their framework structure.^13,14^ Similarly, zeolitic imidazolate framework (ZIF) due to their structural diversity and performance superiority have been widely used in the CO_2_ adsorption process.^15^ Despite that it also has some disadvantages such as poor thermal and chemical stability and the material production cost is also not economical. Activated carbon (AC) has a high surface area and AC is also used as the solid adsorbent for CO_2_ capture.^16^ But AC has few limitations in CO_2_ capture due to its poor thermal stability at high temperatures. Mesoporous silica materials are broadly used in industrial gas separation applications. The presence of channels in the mesoporous silica framework structure helps in the rapid gas diffusion process.^9^ The attraction of CO_2_ towards the silica surface is low compared to the attraction of CO_2_ by other solid adsorbents such as MOF and zeolite and this is due to the absence of cationic sites in the mesoporous silica framework structure.^2,9^

Currently, many researchers are trying to overcome the above problems by using high adsorption and better CO_2_ selective process and economically viable materials.^17^ The zeolite a solid adsorbent is considered to be a good material due to its framework construction which helps for a better selective CO_2_ adsorption process.^18^ Zeolites are well known for their high thermal and chemical stability, better ion exchange properties and have the advantage of variable Si/Al ratio which make zeolites suitable for high CO_2_ adsorption.^19,20^ Zeolites are crystalline microporous solids and consist of tetrahedral TO_4_ (T = Si, Al) units connected to the oxygen atoms present in it. They are crystalline inorganic materials composed of alkali and alkaline earth metal cations with hydrated aluminosilicates forming a three-dimensional framework structure. The aluminosilicates in the tetrahedral framework have the capability of generating porous structure having wide variety of cavities and channels, containing dimensions up to 2 nm. The tetrahedral atoms (T = Si, Al) shared *via* oxygen atoms are considered as primary building block unit (PBU), the repeated unit of PBU combines to form secondary building block units (SBU) which give rise to the three-dimensional zeolite framework structure.^21^ Though zeolites occur in nature, synthetic zeolites have caught great attention in the last decade because it can be produced with different Si/Al ratio and can be obtained with high purity when compared to natural zeolite. By altering the environmental aspects such as temperature and time during the synthesis it is possible to obtain zeolites with different Si/Al ratio.^22,23^ Additionally, the structural arrangement allows modification in the physicochemical properties such as the chemical composition, morphology, hydrophobicity, and hydrophilicity. Zeolites are utilized in various fields such as catalysis, gas separation and purification, cation exchange, environmental remediation, food industry, cosmetics, medicine, magnetism and microelectronics.^24,25^

One of the most significant disadvantages of zeolite synthesis procedure is the high cost of the reactants used for their preparation. The silica sources used such as fumed silica, colloidal silica, silicic acid, and silicon alkoxides are environmentally hazardous and expensive.^26,27^ In the literature, it is reported that synthesis of zeolite used in industry using the above mentioned silica source may cost up to 40% of the total production cost.^28^ Hence, alternative low cost and environment-friendly silica sources such as clay minerals (smectite, sepiolite, kaolin, palygorskite, attapulgite, vermiculite, *etc.*), fly coal ash, diatoms, and silica extracted from rice husk (RH) are considered to be promising silica sources.^28,29^ The major countries producing paddy rice are China, India, Vietnam, Thailand, United States, and Pakistan. It is also found that approximately 20% weight of this value corresponds to RH, which is considered as the major agro-industrial waste produced worldwide. RHs are composed of 13–29% inorganic compounds, 71–87% of organic materials (lignocellulosic, cellulose, *etc.*), and their elemental composition mainly depends on the kind of rice plant, climate, and soil state. In RH the major inorganic component is silicon dioxide (SiO_2_) approximately 89–97% and the remaining are alkali metal trace elements. Generally, RH is converted into Rice Husk Ash (RHA) by burning and then the ash can be used to produce silicon-based materials.^28,30^ So, RH can be successfully used as the alternative silicon source in zeolite synthesis.

Over the past decades, the research towards the synthesis of zeolite from rice husk ash which is used as silicon source has been widely increased because of the interest to move towards cheaper and green synthesis methods. Halimaton *et al.*^31^ have synthesized the zeolite from rick husk silica with a controlled and uncontrolled thermal process of rice husk and have studied their properties. Gargiulo *et al.*^32^ have prepared zeolite NaX from rich husk ash and they have studied the CO_2_ adsorption properties of the prepared zeolite. Klunk *et al.*^33^ have stated about the addition of different types of external source aluminium (ESA) during the zeolite ZSM-5 synthesis from RHA and have compared their properties with the properties reported by other researchers. Khemthong *et al.*^34^ have synthesized zeolite NaY of pure phase from Rice husk silica (RHS) by two different synthetic routes, one-step and two-step process and have reported about the effect of crystallization time. Mesoporous ZSM-5 zeolites have been prepared by Wang *et al.*^35^ using rice husk ash and then impregnated polyethylenimine (PEI) in it for amine modification and have studied about their CO_2_ adsorption nature at different temperatures.

The reported results show that many researchers have synthesized zeolite by different methods using rice husk, but the surface area of the synthesized zeolites are found to be on the lower side and their CO_2_ adsorption performance is also observed to be low. The focus of the present study is to synthesize zeolites with larger surface area and hence increase the CO_2_ adsorption performance. Herein, we have synthesized zeolites using rice husk ash which is the silicon source and have studied their CO_2_ adsorption performance. According to, a survey by the Food and Agriculture Organisation (FAO), it was found that 759.6 million tons of paddy rice was produced globally in 2018 but the rice husk obtained were dumbed or burned as waste during rice production processing.^36^ So, in the present study we have made an attempt to produce quality zeolite using rice husk ash.

Generally, in zeolite synthesis, an organic template agent is used as a structure-directing agent (SDA). The organic reagents such as ethylene diamine derivatives, tetraethylammonium bromide, tetrapropyl-ammonium hydroxide, silylated polymers, crown ethers, and amphiphilic organosilane are widely used in zeolite synthesis.^37^ The major disadvantages of using organic template agent are it is not environment friendly and is non-economical and it also requires additional heat treatment to remove the template agent.^26,38^

To our knowledge, this study is one among the few reports on the preparation of zeolite NaA and NaX with a large surface area from rice husk ash and also having a better CO_2_ adsorption performance. The main results of the present study are (i) conversion of the waste rice husk into an environmentally friendly silicon source and used it for the synthesis of zeolites. (ii) Zeolites with good crystallinity and controlled size have been synthesized *via* template-free hydrothermal method without using any organic template agent. (iii) The physicochemical properties of as-synthesized zeolite RA and RX have been studied. (iv) The CO_2_ sorption performance were also studied for the synthesized zeolites and the obtained experimental results were fitted by a non-linear curve fit using Langmuir, Freundlich and Toth adsorption isotherms.

## 2. Experimental section

### 2.1 Reagents and materials

Rice husk (RH) was collected from the local rice mill at Coimbatore, Tamil Nadu, India. It is used as an alternative silicon source. Sodium hydroxide (NaOH ≥98.0%, Sigma Aldrich) pellets purchased from Sigma Aldrich India are used as a sodium source and as a counter ion in zeolite synthesis. For aluminium source sodium aluminate (Na–(Na_2_O): 37–45%; Al–(Al_2_O_3_): 50–56%, Sigma Aldrich), is used as an additional sodium and aluminate source. Hydrochloric acid (HCl, 36.5–38.0%) and highly purified double distilled (DD) water were also used during the synthesis process.

### 2.2 Sodium silicate extraction from rice husk

**Fig. 1:**
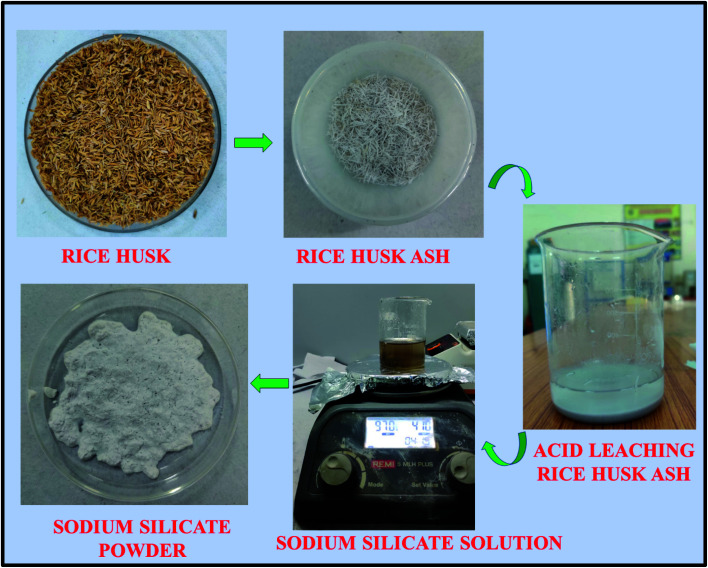
Synthesis procedure of sodium silicate from Rice Husk Ash (RHA).

Rice husk received from the rice mill, were initially washed with tap water followed by DD water to remove the adhering soil and dust present in it. The sodium silicate synthesis from the RH is shown in Fig. 1. The washed rice husk was dried at 110 °C for 8 h in the oven to remove the moisture from it. The dried RH was crushed and milled to homogenize the particle size before the heating process. The homogenized RH was kept in the muffle furnace at 600 °C for 6 h to convert the RH into the rice husk ash (RHA). The metal impurities present in the converted RHA are eliminated by heating the ash in an acidic solution (1 M HCl) at 80 °C for 1.5 h. Then, the resultant mixture was cooled to room temperature and filtered using Whatman filter paper (grade no. 42). The obtained residue is washed with DD water around 6–8 times to make it neutral and dried at 110 °C in an oven for 8 h. The dried powder is collected and crushed using agate mortar. Then, 10 g of material is stirred with 100 ml of sodium hydroxide solution (2.5 M) for about 5 h at 90 °C. Finally, the sodium silicate solution was filtered by using Whatman filter paper (grade no. 42) followed by washing with boiling water and the sediment is dried at 105 °C in an oven for 6 h.^39^ The obtained dried sodium silicate powder was used as alternative source material for zeolite synthesis. The reaction mechanism corresponding to the formation of sodium silicate from the rice husk ash silica is given below.^38^

1 SiO_2_(RHA) + 2NaOH → Na_2_SiO_3_(soluble) + H_2_O

#### 2.2.1 Mechanism

The reaction steps involved in the formation of zeolites (A and X) are given in eqn (2) and (3),^40^2
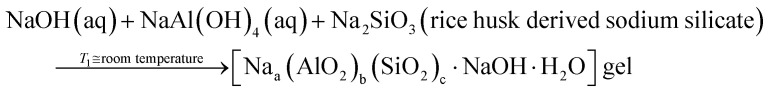
3
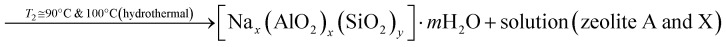


According to eqn (2), the reaction between sodium hydroxide, sodium aluminate and sodium silicate derived from rice husk ash in aqueous solution at room temperature results in the formation of amorphous gel. The gel formation is mainly attributed to the copolymerization of the silicate and aluminate species by the condensation – polymerization mechanism. From eqn (3), it is observed that the crystalline phase of the gel is achieved by the hydrothermal autogenous condition taking place at temperatures of 90 °C and 100 °C for zeolite X and A respectively and the detailed procedure for synthesis of zeolites are given below.

#### 2.2.2 Synthesis of zeolite NaA (RA)

Zeolite NaA type was synthesized without using an organic template agent by hydrothermal method using sodium silicate extracted from RHA as a silicon source. The molar ratio of the initial gel used for synthesis was 2NaO_2_ : Al_2_O_3_ : 1.96SiO_2_ : 132H_2_O.^41^ Typically, two identical solutions with 40 ml of DD water and 0.385 g of NaOH were prepared separately and they were named as A and B. In the A solution, the calculated amount of 7.26 g of sodium aluminate was added and stirred for 15–20 minutes. Simultaneously, in the B solution, the required amount of 22.49 g sodium silicate extracted from RHA was added and stirred for 15–20 minutes. After stirring the two solutions separately for a certain time a homogeneous mixture was produced, then the solution B is quickly transferred to A solution and the gel was stirred vigorously for 30 minutes. Then the resultant mixture was transferred to the Teflon lined stainless steel autoclave and heated up to 100 °C for 8 h. Finally, the products are recovered by centrifugation process and the obtained residue was dried at 80 °C for overnight. The solid white powder obtained using RHA was named as zeolite NaA (RA).

#### 2.2.3 Synthesis of zeolite NaX (RX)

The compositional ratio used for the synthesis of zeolite NaX is 16NaO_2_ : Al_2_O_3_ : 4SiO_2_ : 325H_2_O and the synthesis method used is also similar to the above-mentioned method.^20^ The A and B solutions were prepared by adding 3.16 g of NaOH to 30 ml of DD water and they were stirred until they get completely mixed. Then 2.87 g of Sodium aluminate was added to the A solution and 4.31 g of sodium silicate extracted from RHA was added to the B solution. After this both solutions were stirred separately for 20 minutes until they were completely mixed and become a homogenous solution. Then, quickly B solution was transferred to the A solution to form a reaction gel and stirred for 45 minutes. The reactive gel was transferred into the Teflon lined autoclave for the crystallization process and heated at 90 °C for 24 h. Then the mixture was recovered by centrifugation process and washed with DD water until the pH was reduced to approximately to 8–9. Finally, the residue was collected after the centrifugation process and was dried in the oven at 80 °C overnight. The obtained solid product was named as zeolite NaX (RX).

#### 2.2.4 Characterizations

The structural properties of RHA, SS and zeolite materials were studied by using X'Pert Pro powder diffractometer (PANalytical, Holland) with Cu (Kα) radiation (*λ* = 1.54 Å), operated at 40 kV and 30 mA in the scanning range of 2*θ* = 5–50° with the step size of 0.02°. The functional groups present in the materials have been identified by using FTIR-Shimadzu IR Affinity model 1 s fitted with a double beam spectrometer. The scanning range is 4000 cm^−1^ to 400 cm^−1^ with a resolution of 0.5 cm^−1^. The morphology of the samples have been studied using Field emission scanning electron microscope (FESEM) (SIGMA HV- Carl Zeiss with Bruker Quantax 200- Z10) fitted along with an energy-dispersive X-ray spectroscopy EDS detector. Further, the higher magnification images were obtained by using JEOL JEM 2100 High-Resolution Transmission Electron Microscope (HRTEM) with lattice resolution of 0.14 nm and point-to-point resolution of 0.19 nm and operating at 200 kV acceleration voltage. The thermal properties of the materials have been studied by using TG/DTA (SII 6300 EXSTAR) integrated with a thermal analyser (TA Instruments) using N_2_ gas atmosphere in the range of 35–900 °C. The N_2_ nitrogen sorption studies at −196.15 °C have been carried out by using an ASAP 2020 Quantachrome Instruments, Autosorb IQ series (Micromeritics, USA). The samples are degassed at 300 °C for 3 h before analysis. The textural properties have been studied by the BET method and the pore size has been calculated by using the BJH equation.

#### 2.2.5 Gas adsorption measurements

The adsorption properties of CO_2_ have been studied by the Belsorp analyser series instrument (BELSORP-max, MicrotracBEL, Japan). Before analysis, the samples are degassed at 300 °C for 3 h. The operating temperature during the measurement of CO_2_ adsorption is 297.15 K. The experimentally obtained data are further studied by a non-linear curve fit using Langmuir, Freundlich and Toth adsorption isotherms.

## 3. Results and discussion

### 3.1 Structural properties of RHA

**Fig. 2:**
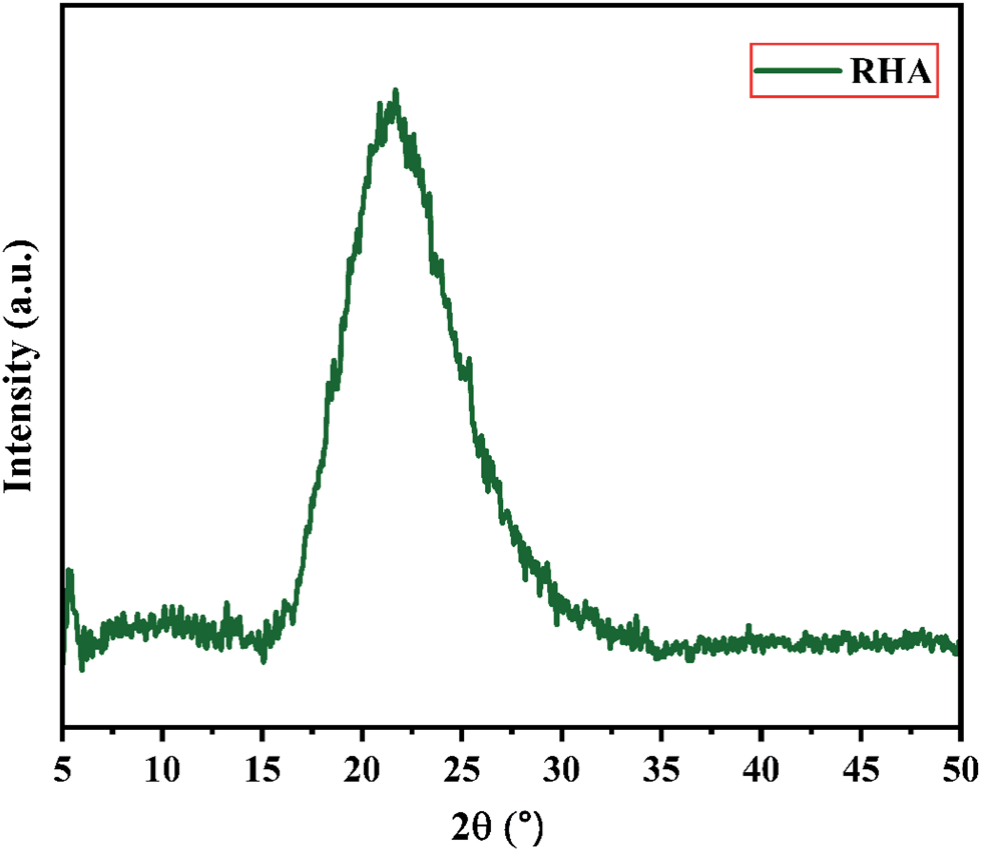
X-ray diffraction pattern of Rice Husk Ash (RHA).

Fig. 2 shows the XRD pattern of the rice husk ash (RHA) sintered at 600 °C. It is observed from the figure that only a broad intense peak of SiO_2_ is obtained at 2*θ* = 21.6° which typically indicates the formation of amorphous silica. There are also no sharp peaks present in the range 2*θ* = 5–50° indicating the absence of any ordered crystalline structure and confirms the amorphous nature of the material.^42^ From the obtained XRD diffractogram, it is clearly understood that RHA contains pure silica in an amorphous state with limited impurities and it is considered as a suitable candidate for the synthesis of sodium silicate.

**Fig. 3:**
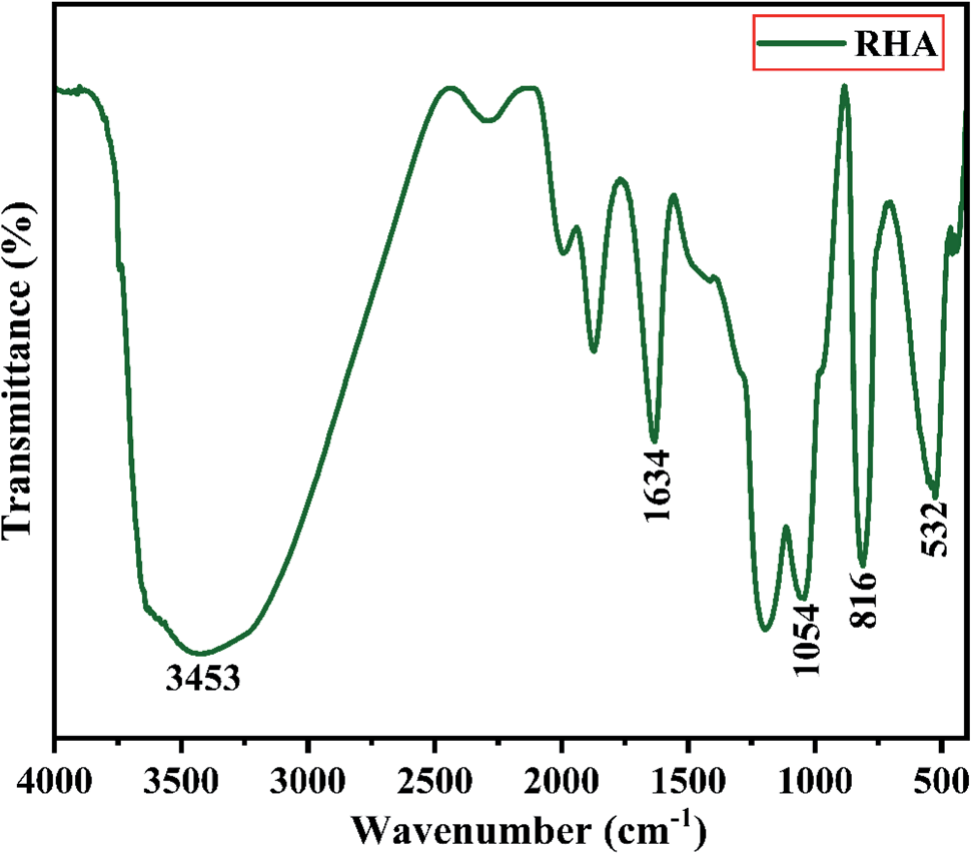
FTIR spectra of Rice Husk Ash (RHA).

Fig. 3 shows the FTIR spectrum of RHA analysed in the 400–4000 cm^−1^ wavenumber range. The broadband observed at 3453 cm^−1^ is attributed to the stretching vibration band of O–H corresponding to water molecules. The peak at 1634 cm^−1^ corresponds to the deformation vibrations of (δ-H_2_O) water molecules. The vibration bands at 1054 and 816 cm^−1^ correspond to the Si–O–Si stretching vibration of RHA. The band at 532 cm^−1^ denotes the O–Si–O bending vibration and is found to be sharper as the organic matter is no longer present in it.^43,44^

**Fig. 4:**
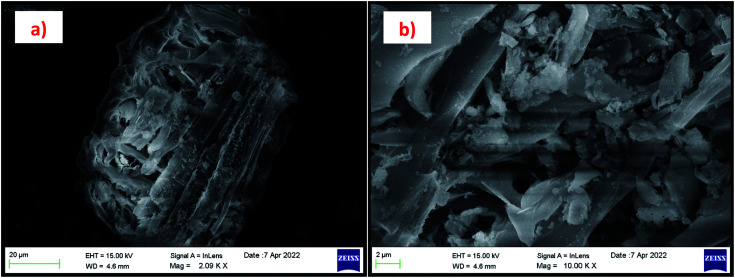
(a and b) FESEM micrograph of RHA at different magnifications.

**Fig. 5:**
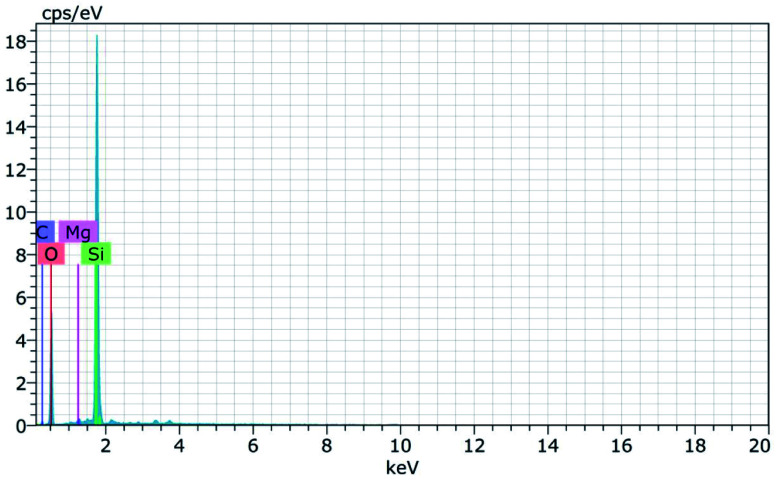
EDAX spectra of RHA.

The morphology of the calcinated RHA is studied by using FESEM analysis. Fig. 4(a) and (b) shows the micrographs of the RHA at different magnifications. From the figure, it is observed that wave-like morphology is observed for RHA. For the calcinated RHA several cracks have been recorded which indicates the crumbliness nature of the sample.^45^Fig. 5 shows the EDAX spectra of RHA. From the spectra, it is found that only elements such as Si, O, and C are the major elements in RHA and a tiny amount of Mg is present in RHA.^46^ The elemental composition of the RHA is given in Table 1.

**Table 1:** Elemental composition of RHA

Elements	Si%	O%	C%	Mg%
RHA	27.78	60.38	11.47	0.38

#### 3.1.1 Structural properties of sodium silicate derived from RHA

**Fig. 6:**
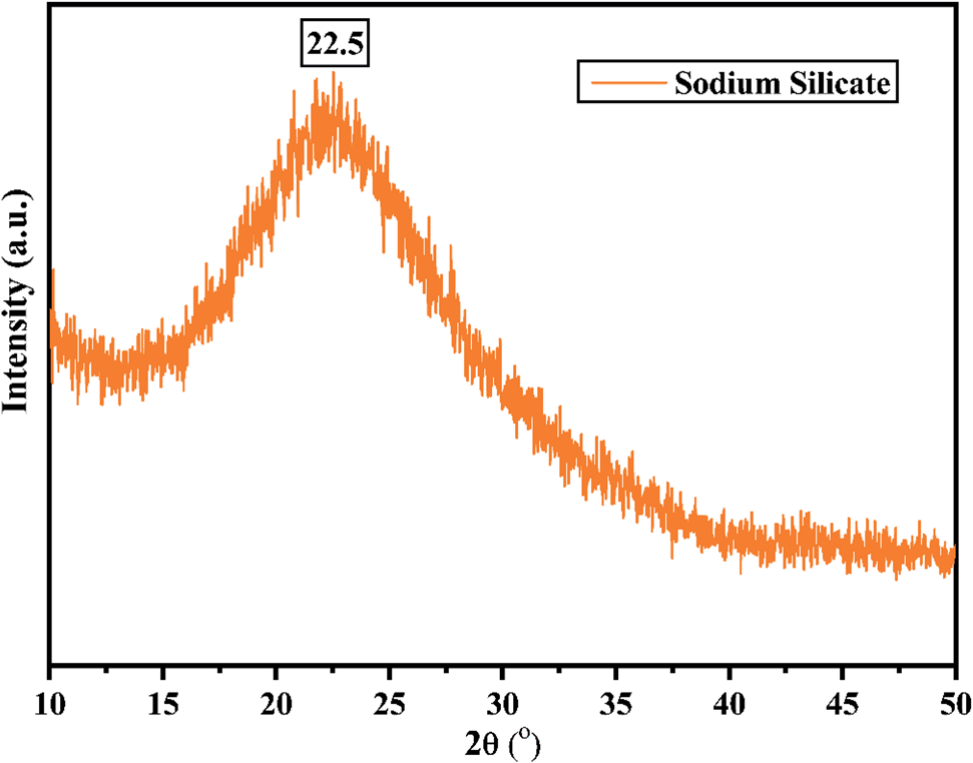
X-ray diffraction pattern of sodium silicate (SS) synthesized from RHA.

**Fig. 7:**
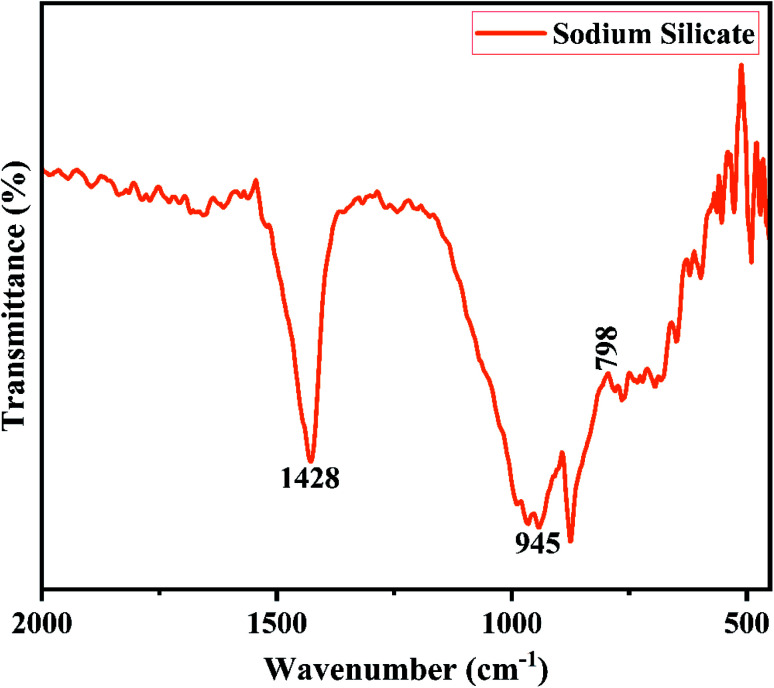
FTIR spectra of SS synthesized from RHA.

The X-ray diffractogram of sodium silicate derived from RHA obtained using 2.5 M of NaOH is shown in Fig. 6. From the figure, it is noted that a broad intense peak is obtained at 2*θ* = 22.5° which corresponds to sodium silicate.^43^ The obtained pattern is not having any sharp peak indicating the formation of amorphous sodium silicate. Fig. 7 shows the FTIR spectrum of the sodium silicate extracted from RHA. The characteristic bands present in the range of 945–1428 cm^−1^ corresponds to the Si–O–Si. The broadband present in the range 480–798 cm^−1^ are the vibrational bands of O–Si–O corresponding to bending vibrations.^43,44^

**Fig. 8:**
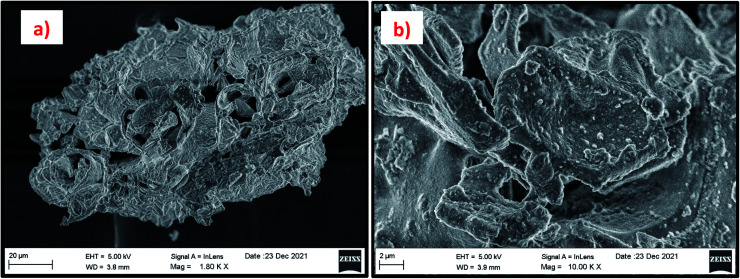
(a and b) FESEM micrograph of SS synthesized from RHA at different magnifications.

**Fig. 9:**
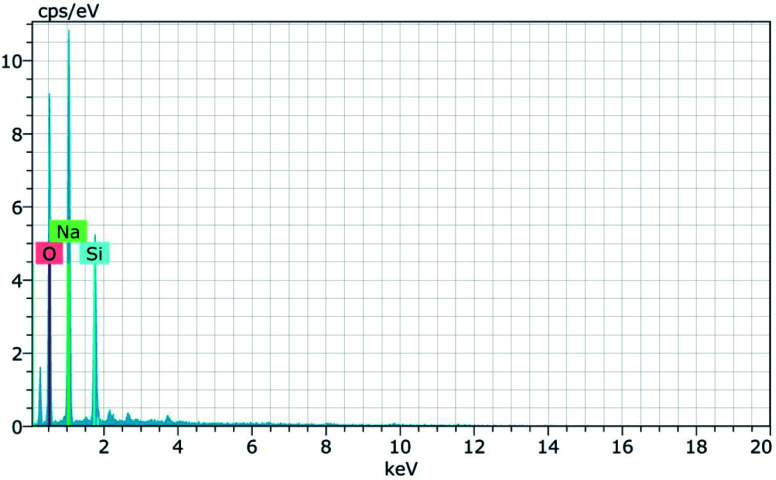
EDAX spectra of SS synthesized from RHA.

Fig. 8(a) and (b) shows the FESEM micrographs of sodium silicate at two different magnifications. From the obtained micrographs it is observed that layer like “flakes” structure is present in the materials.^43^ The elemental composition of the synthesized sodium silicate is found using EDAX spectral analysis and is shown in Fig. 9. From the spectrum, it is observed that the elements Na, Si, and O are present in the synthesized sodium silicate. Table 2 shows the weight percentage of the elements present in sodium silicate obtained from RHA.^42,43^

**Table 2:** Elemental composition of SS synthesized from RHA

Elements	Na%	Si%	O%
Sodium silicate	29.54	10.65	59.81

### 3.2 X-ray diffraction (XRD) analysis

**Fig. 10:**
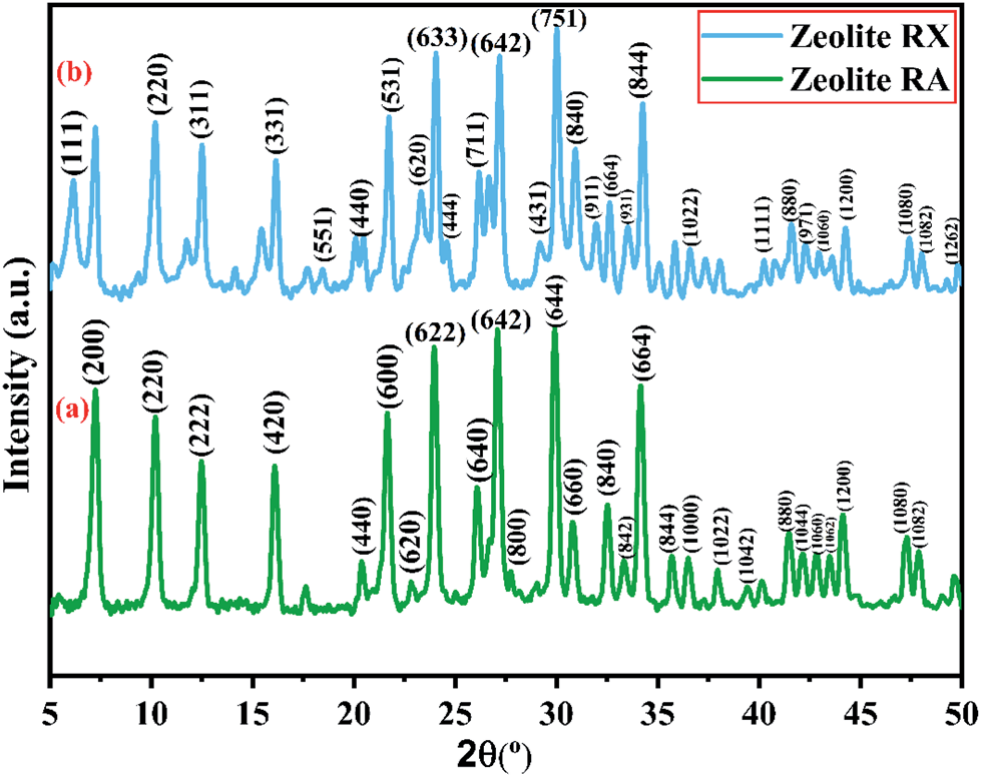
(a and b) X-ray diffraction of zeolite RA and RX synthesized from RHA.

X-ray powder diffractogram has been recorded for the synthesized zeolite to know about the type of the zeolite and its purity. Fig. 10(a) and (b) shows the X-ray diffraction pattern of the synthesized zeolite RA and RX which has strong peaks in the range of 2*θ* = 5–35°. The presence of sharp peaks in the diffraction pattern indicate that the synthesized material is crystalline in nature and the peaks have been indexed and are found to match well with the reported results.^47,48^

Fig. 10(a) shows the XRD diffraction pattern of the synthesized zeolite RA. The diffraction peaks are present at 2*θ* = 7.2°, 10.2°, 12.4°, 16.1°, 20.3°, 21.6°, 23.9°, 26.0°, 27.1°, 30.7°, 32.5°, 33.3°, 34.2°, 35.7°, 36.4°, 37.9°, 39.4°, 41.4°, 42.1°, 42.9°, 43.5°, 44.1°, 47.2° and 47.9° indicating that the synthesized material exhibits good crystallinity. The obtained pattern is compared with the standard Joint Committee on Powder Diffraction Standards (JCPDS) card # 39-0222, Na_96_Al_96_Si_96_O_96_·216H_2_O ∼ Si : Al : Na = 1 : 1 : 1 and is found that the major phase in the synthesized sample is zeolite A type. The synthesized zeolite is found to exhibit face-centred cubic crystal structure. The synthesized material belongs to the *Fm*3̄*c* space group and cell parameters are found to be *a* = *b* = *c* = 24.61 Å.^41,47^

Fig. 10(b) is the X-ray diffractogram of the prepared sample zeolite RX. It can be seen from the figure that typical diffraction peaks are obtained at 2*θ* = 6.1°, 10.2°, 12.4°, 16.1°, 18.4°, 20.4°, 21.7°, 23.3°, 24.0°, 24.6°, 26.1°, 27.1°,29.2°, 30.0°, 30.9°, 31.9°, 32.6°, 33.4°, 34.2°, 36.5°, 40.3°, 41.6°, 42.1°, 42.2°, 42.9°, 44.2°, 47.3°, 48.0° and 49.8° and the sample is crystalline in nature. The recorded XRD peaks are compared with JCPDS card # 38-0237, Na_86_Al_86_Si_106_O_384_ : 260H_2_O ∼ Si : Al : Na = 1.2 : 1 : 1 and are found to match well with the JCPDS card suggesting that the material is zeolite X type. The material belongs to the cubic structure and lattice is found to be face centred in nature. The lattice parameters that have been calculated and are *a* = *b* = *c* = 24.99 Å.^48,49^

According to the hydrogel formulae used for the synthesis of zeolite RA and RX, a higher concentration of silica is required for the formation of more complex Double Six Rings (D6R) of zeolite RX, and it also requires more energy and time for formation when compared to the formation of zeolite RA. From the obtained XRD data the average crystallite size of the prepared material is calculated using Debye–Scherrer's relation.4
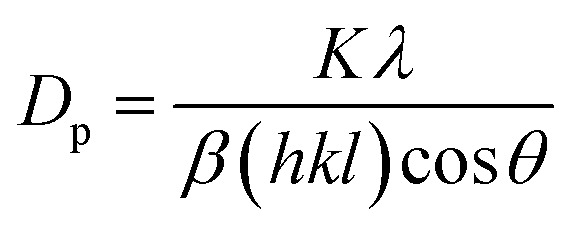
where *K* is the Scherrer's constant (*K* = 0.9), *λ* is the wavelength of the X-ray used (*i.e.*, *λ* = 1.5406 Å). *β*(*hkl*) represents the full width at half maximum (FWHM) and 2*θ* is the angle of incidence of X-rays. Using the above equation, the average crystallite size of the prepared samples have been calculated and are found to be 37.93 nm, and 24.02 nm respectively.

#### 3.2.1 Fourier transform infrared spectroscopy (FTIR)

**Fig. 11:**
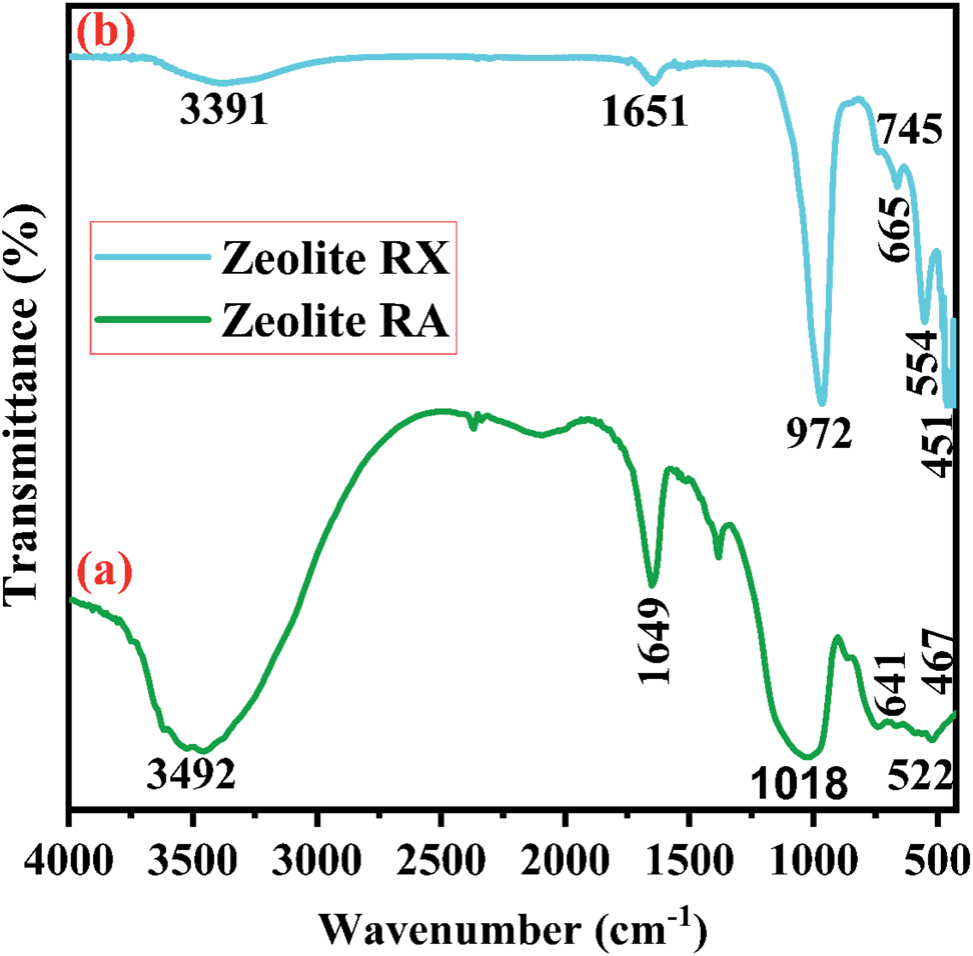
(a and b) FTIR spectra of zeolite RA and RX synthesized from RHA.

FTIR spectra are used to study the functional groups associated with the material and each functional group has its own discrete vibrational energy which can be used to identify the molecule. Fig. 11(a) and (b) are the FTIR spectra of synthesized zeolite RA and RX respectively. In Fig. 11(a) the peak at 467 cm^−1^ corresponds to the O–Si–O bending vibration of the material. A broad peak of low intensity observed at 522 cm^−1^ indicates the vibration band of double four rings (D4R) and is observed due to the interrelation of symmetrical stretching vibration of tetrahedron (T–O–T) of Si–O–Si bonds and this peak indicates the existence of zeolite NaA band.^50^ The vibration band located at 641 cm^−1^ corresponds to the internal vibration of the symmetrical stretching vibration of Al–O–Si vibrations. The band observed at 1018 cm^−1^ is due to the as-symmetrical stretching vibration of T–O–Si (T: Al or Si) present in the aluminosilicates of the hydrogel. The band at 1649 cm^−1^ is due to the presence of scissor-type bending vibrations of the adsorbed water molecules. The vibration band at 3492 cm^−1^ is attributed to the structural OH stretching vibrational band of prepared zeolite RA hydrogel.^50,51^

Fig. 11(b) is the FTIR spectra of the synthesized zeolite RX. The band at 451 cm^−1^ is due to the as-symmetrical stretching vibrations of Si–O–Al tetrahedra bonds of the hydrogel. The vibrational band at 554 cm^−1^ corresponds to the symmetrical vibrations of Si–O–Si bonds and is obtained due to double six rings (D6R) which interconnects the sodalite cages to form zeolite RX.^51^ The vibration bands present at 745 and 672 cm^−1^ denote the symmetrical stretching of internal T–O–T (T = Si or Al) tetrahedral and external T–O–T linkage of symmetrical stretching vibrations of the prepared hydrogel. The symmetrical stretching vibration of the Si–O–Si tetrahedral bond is observed at 972 cm^−1^. The absorption band at 1651 cm^−1^ corresponds to the bending vibration of internal water molecules. The band at 3391 cm^−1^ represents the stretching vibrational band (H–O–H) of the adsorbed water molecules present in the synthesized zeolite RX.^20,51^ The bands observed for the synthesized zeolites are indexed and their respective band values are found to match well with the values reported in the literature.^50,51^

#### 3.2.2 Thermal analysis (TGA/DTG/DTA) studies

**Fig. 12:**
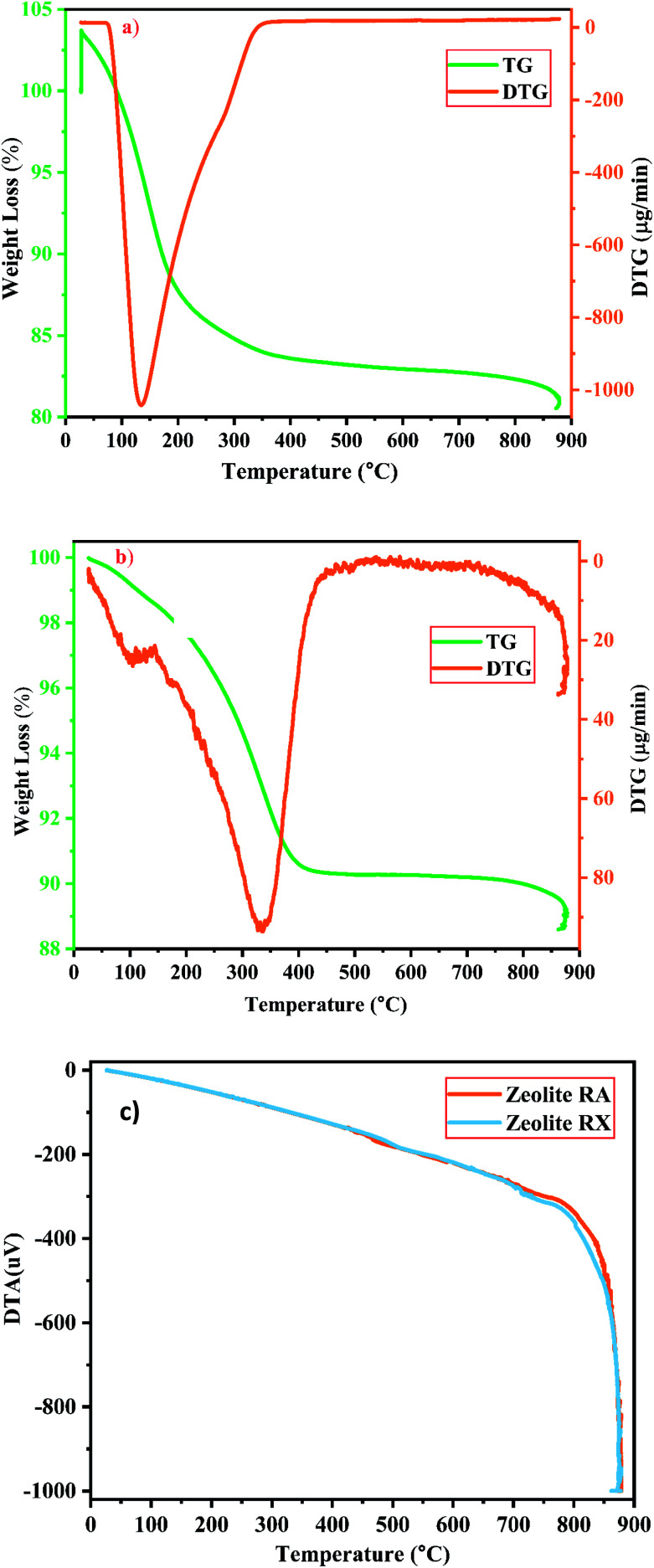
(a and b) TG/DTG investigation of zeolite RA and RX and (c) DTA spectra of zeolite RA and RX synthesized from RHA.

The thermal properties of the prepared materials (*i.e.*, thermal stability, weight loss, *etc.*) are investigated by using thermal analysis methods at elevated temperature under various atmospheres. It is observed that during the thermal analysis process of the zeolites, the weight loss is mainly observed in two forms in the structure and is mainly due to the reduction and discharge of water molecules present in it. On the surface of the material and cavities present in the material, the water molecules are present in the form of moisture, the mass loss occurs at low temperature in the range around 100–150 °C. At higher temperature, the mass loss occurs due to the elimination of water molecules present in the zeolite framework structure that bonds with the neighbouring molecules, without causing any disturbance to the framework structure of the prepared materials.^25^ The TGA/DTA analysis has been used to study about the mass loss and other thermal properties of the sample. Fig. 12(a) and (b) are the graphs corresponding to the TGA/DTG studies of the prepared zeolites RA and RX carried out in the presence of N_2_ atmosphere in the temperature range of 25–880 °C respectively.

For the synthesized zeolite RA and RX, it is noted that the preliminary weight loss starts at 25 °C and loss continues approximately up to 300 °C and it occurs due to the loss of water molecules present in the form of moisture and volatiles within the pores. In this temperature range the weight loss in the samples of RA and RX are approximately 15% and 5% respectively^25,52^ and are shown in Fig. 12(a) and (b). The loss is obtained due to the presence of water molecules at different energy levels in the zeolite lattice structure and it depends on the type of coordinated cations present in it. The water molecules present in the zeolite structure is linked to the water in such a way that it can easily move into and out of the zeolite lattice without disrupting the framework structure. However, for temperatures above 200 °C the mass loss of the zeolite is attributed to the moisture loss which is due to the breaking of hydration complex molecules with exchangeable cations present in the framework of zeolite structure.^25^

In the DTG curves shown in Fig. 12(a) and (b) the presence of desorption peak at 135 °C for zeolite RA and 335 °C for zeolite RX indicate the existence of one type of water grafting loss in the framework structure and represents one type of bonding.^25,38^ The second minimal weight loss for both the samples is continuous up to 800 °C and it occurs due to the hydroxylation process which causes the destruction of hydroxyl bonds with cations that leads to the removal of excess water molecules present in the zeolite framework cavities. At this stage the maximum amount of weight loss obtained for the synthesized zeolite is approximately about 4%.^25^

Fig. 12(c) shows the DTA curve of the synthesized zeolite RA and RX. From the DTA curve, it is found that the endothermic region is observed around the range of 200–400 °C for the zeolite RA and RX.^25,50^ This is due to the loss of water molecules existing at the surface of the samples. For the synthesized zeolite materials RA and RX, it is observed that the weight loss occurs gradually in the temperature range of 400–800 °C. The presence of exothermic peaks at higher temperature range for the samples are linked to collapse in the framework structure of the zeolite. At higher temperatures, the exothermic peaks are also helpful for studying the thermal stability of the materials.^53^ However, the thermal stability of the synthesized zeolite is directly proportional to the Si/Al ratio of the material. From the obtained results, the zeolite RX with a high Si/Al ratio is found to have high thermal stability when compared to zeolite RA.

#### 3.2.3 Field emission scanning electron microscopy (FESEM) and energy dispersive X-ray analysis (EDAX) results

**Fig. 13:**
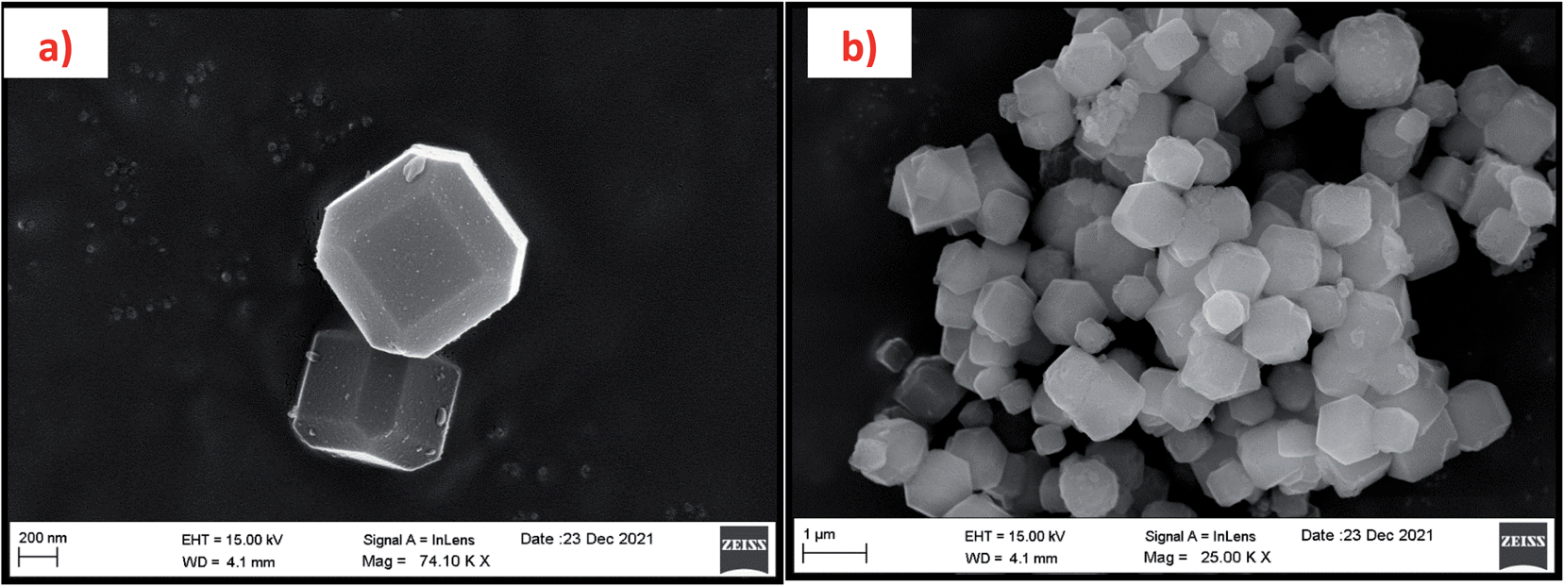
(a and b) FESEM micrographs of zeolite RA synthesized from RHA at two magnifications.

**Fig. 14:**
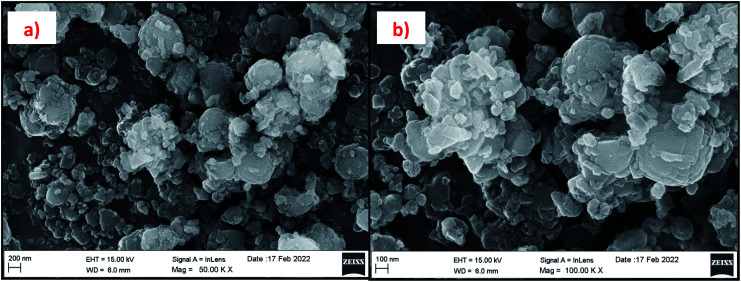
(a and b) FESEM micrographs of zeolite RX synthesized from RHA at two magnifications.

The morphology of the synthesized zeolites is studied by using FESEM analysis. Fig. 13 and 14(a) and (b) are the micrographs of synthesized zeolite RA and RX at different magnifications. For each synthesized zeolite, two designated micrographs are selected to illustrate the structure of the material. Fig. 13(a) and (b) show the zeolite RA micrographs which suggest that the samples exhibit an “Ice cube” morphology with chamfered (rounded-off corners) edges.^50^ The secondary building block unit (SBU) of the sodalite cage (β-cage) is connected with D4R to form the three-dimensional (3D) micropore cubic structure (α-cage) morphology.^54^ The particle size of the zeolite RA is found to be about 0.6–0.9 μm with an average diameter of 0.8 μm. Fig. 14(a) and (b) are the images of zeolite RX recorded at two different magnifications. The micrographs show that the particles exhibit uniform size distribution having octahedral morphology with an average diameter of about 1–2 μm.^20^ Similarly, the repeated unit of SBU sodalite cage (β-cage) is linked with D6R to form the octahedral morphology (α-cage).

**Fig. 15:**
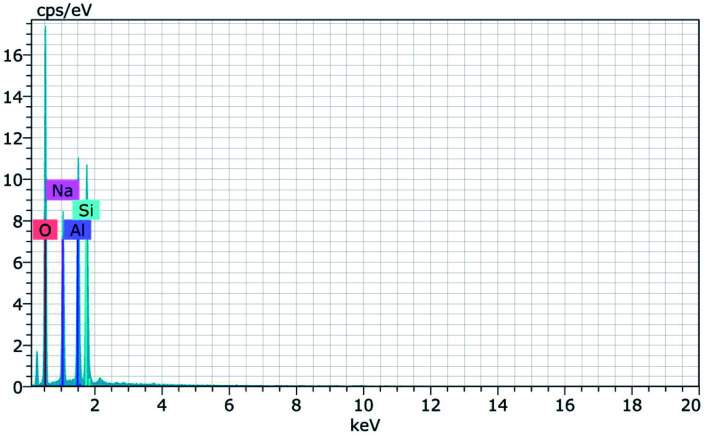
EDAX spectra of zeolite RA synthesized from RHA.

**Fig. 16:**
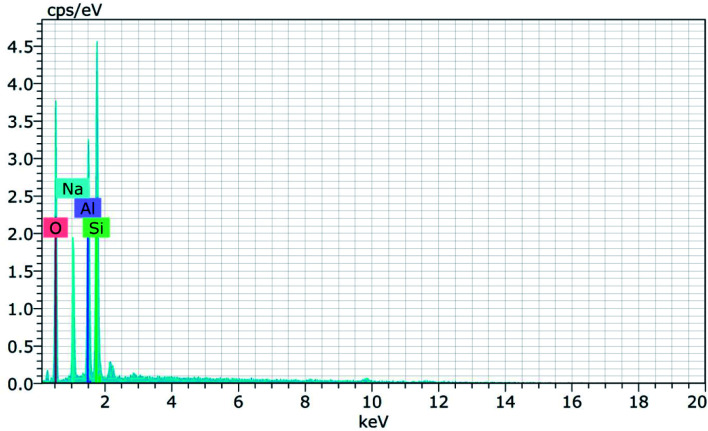
EDAX spectra of zeolite RX synthesized from RHA.

Fig. 15 and 16 show the EDAX spectra of the synthesized zeolite RA and RX. From the spectra, it is observed that only elements such as Na, O, Si and Al are present in the samples. The chemical composition of the synthesized zeolites is given in Table 3. The Si/Al ratio plays an important role in the zeolite synthesis process.^50,55^ For the zeolite RA the Si/Al ratio is found to be approximately 1 and for zeolite RX it is about 1.27. Additionally, the elemental mapping of the synthesized zeolite RA and RX are given in the ESI Fig. S1 and S2.† The micrographs show the presence of Na, O, Si and Al elements and they are found to be evenly distributed in the synthesized zeolites.

**Table 3:** Elemental composition of synthesized zeolite RA and RX from RHA

Zeolites	Na%	Si%	Al%	O%	Si/Al
Zeolite RA	15.94	14.79	15.34	53.93	0.96 (∼1)
Zeolite RX	13.41	21.06	15.89	49.64	1.32

#### 3.2.4 High-resolution transmission electron microscope (HR-TEM) studies

**Fig. 17:**
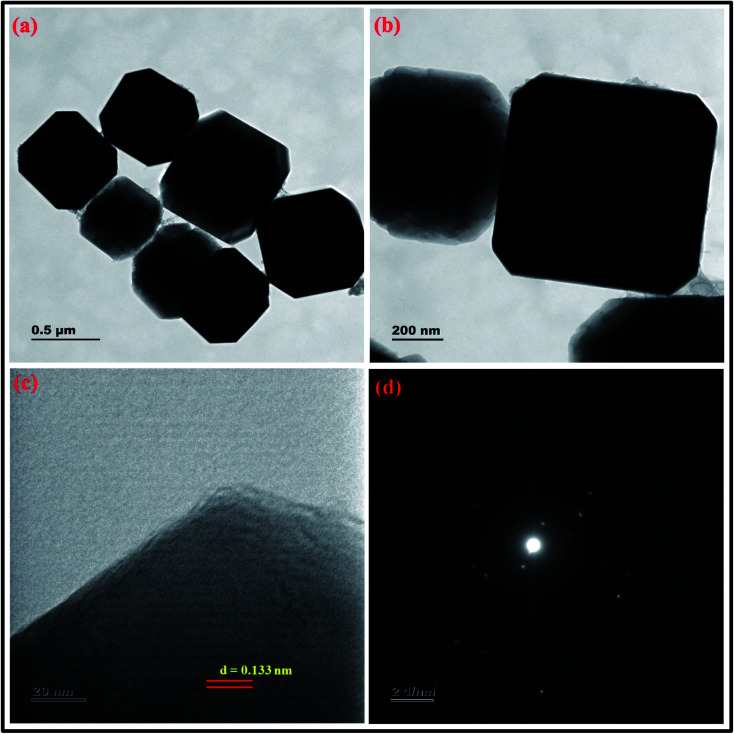
(a and b) HRTEM micrographs at two magnifications (c) lattice fringes with *d*-spacing value (d) SAED pattern of zeolite RA synthesized from RHA.

**Fig. 18:**
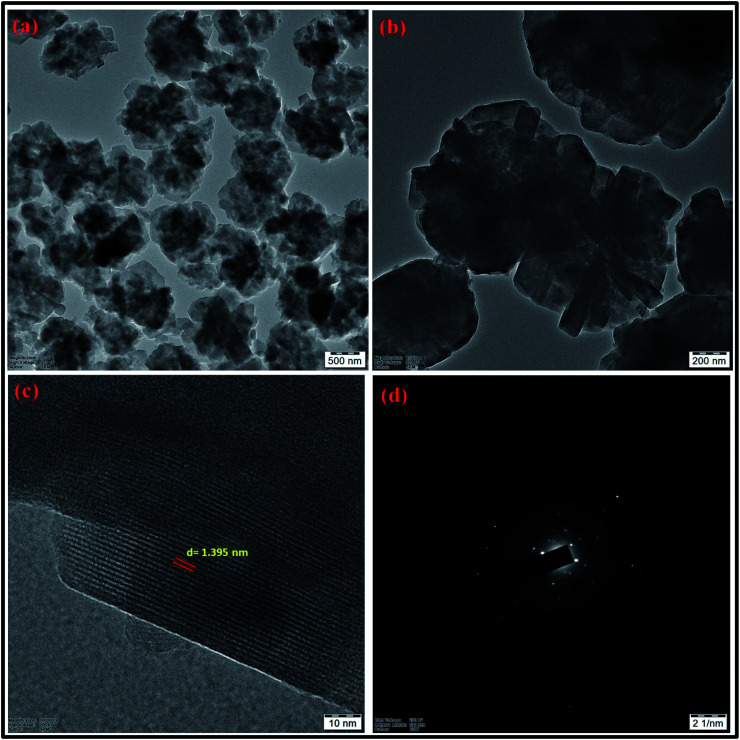
(a and b) HRTEM micrographs at two magnifications (c) lattice fringes with *d*-spacing value (d) SAED pattern of zeolite RX synthesized from RHA.

The crystal structure of the synthesized zeolite RA and RX have been studied using HRTEM analysis. Fig. 17 and 18(a)–(d) show the HRTEM images of the synthesized zeolite RA and RX. Fig. 17(a) and (b) shows the HRTEM micrographs of zeolite RA recorded at two different magnifications. The images indicate that the samples exhibit an “Ice cubic” structure which confirms the cubic nature of the materials.^56^ The average particle size of the material is about 0.6–0.8 μm. The lattice fringes of the zeolite RA are shown in Fig. 17(c). From the lattice fringes, the *d* spacing value is calculated and is about 0.133 nm and corresponds to the peak (644) of zeolite RA. Fig. 17(d) shows the SAED pattern of the zeolite RA and the pattern has spots arranged in a particular manner which indicates the polycrystalline nature of the synthesized material.^56,57^Fig. 18(a) and (b) are the HRTEM images of the synthesized zeolite RX at different magnifications. It reveals that the particles are of octahedral shape with uniform size.^56^ The particle size of the synthesized zeolite is found to be in the range of 0.9–1.2 μm. Fig. 18(c) shows the lattice fringes of the zeolite RX and they are found to be orientated in the same direction. From the lattice fringes, the *d* spacing value is calculated and is 1.395 nm corresponding to the (111) plane. Fig. 18(d) shows the SAED pattern of the synthesized zeolite RX and the pattern indicates that the synthesized material is of polycrystalline nature.^56^

#### 3.2.5 N_2_ adsorption–desorption studies

**Fig. 19:**
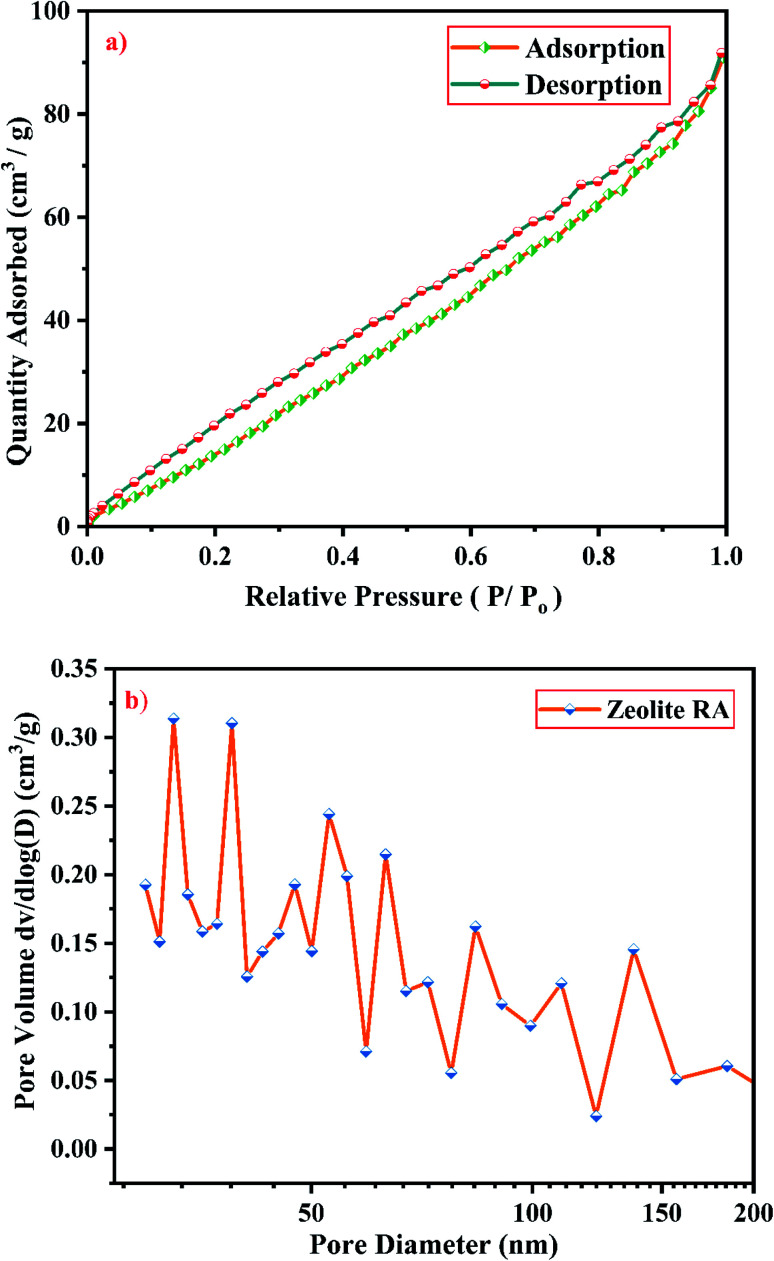
(a) N_2_ sorption studies and (b) BJH equation plot of zeolite RA synthesized from RHA.

**Fig. 20:**
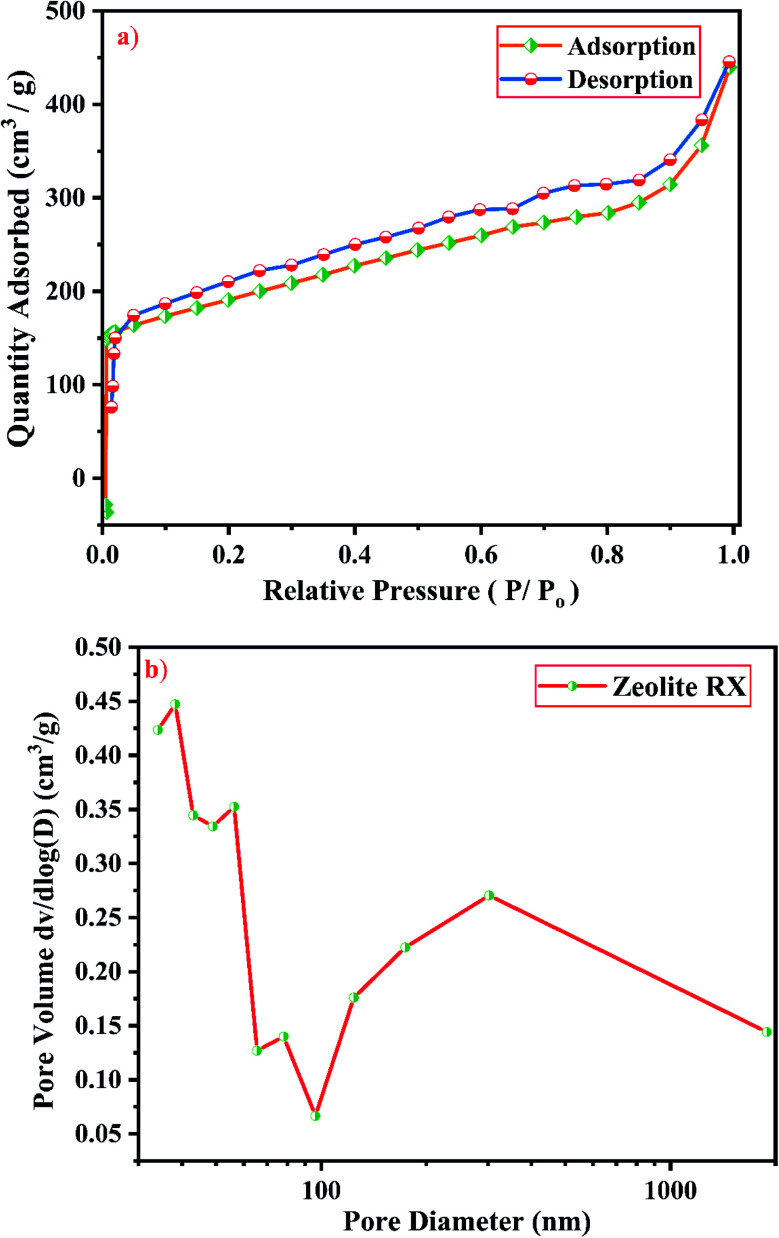
(a) N_2_ sorption studies and (b) BJH equation plot of zeolite RX synthesized from RHA.

Knowledge of surface area, volume and pore size distribution of the synthesized material is required to use it as catalysts and adsorbents. BET method (calculated by the quantity of nitrogen gas adsorbed and desorbed by a material at constant liquid nitrogen temperature −196 °C) is used to measure accurately the total pore surface of the samples.^58^Fig. 19(a) and 20(a) show the N_2_ adsorption–desorption isotherms of zeolite RA and RX respectively. It is mandatory to mention that the surface area of zeolite RA cannot be measured accurately by nitrogen sorption studies due to its smaller pore size (4 Å) and due to the presence of Na^+^ cation which occupies the position near the micropore aperture. At low relative pressure (<0.1), there is a sharp increase in N_2_ adsorption in the zeolite RA and it also shows that N_2_ molecules reside at the pore opening of the framework structure.^59^

From Fig. 19(a), it is observed that a typical type IV adsorption isotherm according to the IUPAC classification criterion is found for the zeolite RA along with the H3 hysteresis loop, which indicates that the material has a mesoporous nature.^38^ Due to its smaller pore size, at low relative pressure there is only a small amount of intake of N_2_ molecules. At high relative pressure (*P*/*P*_o_ = 0.9–1.0), there is a significant increase in the adsorption indicating that there are large number of mesopores present in the sample. For zeolite RX, due to the single layer adsorption of N_2_ molecule there is a sharp rise in the curve below *P*/*P*_o_ = 0.03 which indicates the filling of the micropores of the sample and is shown in Fig. 20(a). A type I adsorption isotherm with H4 hysteresis loop is obtained for the zeolite RX according to IUPAC classification.^25,49^ In the pressure range between *P*/*P*_o_ = 0.4–0.8, a slit-shaped curve is observed due to the filling of mesoporous network in the obtained zeolite framework structure. Due to the capillary condensation of nitrogen gas and filling of mesopores at the high relative pressure *P*/*P*_o_ > 0.9, a weak desorption hysteresis loop curve is obtained.^25^ The BET surface areas are 106.25 m^2^ g^−1^ and 512.79 m^2^ g^−1^ for the synthesized zeolite RA and RX respectively. The kinetic diameter of the N_2_ molecule is 0.36 nm, it is similar to the pore size of zeolite RA (about 0.4 nm) at the temperature of −196 °C so it is unable to enter into the pore of zeolite RA structure. So, zeolite RX has a higher surface area and pore volume compared to zeolite RA.

Fig. 19(b) and 20(b) shows the pore size distribution of the synthesized zeolite RA and RX respectively. The pore size distribution is calculated by using Barrett–Joyner–Halenda (BJH) equation. For zeolite RA the majority of the pores have a size lying in the region between 30-50 nm with the average pore size of 32 nm as shown in Fig. 19(b). Fig. 20(b) shows the pore size distribution of zeolite RX. It is found that most of the pores are having a size lying in the region between 35–50 nm with an average pore diameter of 38 nm.^20,38^ For both the synthesized zeolites the average pore size diameter is found to be in the mesoporous region according to the IUPAC classification criterion. Table 4 gives the BET surface area and pore volume of synthesized zeolite RA and RX.

**Table 4:** Comparison table of textural properties of zeolite RA and RX synthesized from RHA

Samples	Silicon source	*S*_BET_ (m^2^ g^−1^)	Total pore volume (cm^3^ g^−1^)	References
Zeolite RA	Rice husk ash (RHA)	106.25	0.139	This work
Zeolite RX	Rice husk ash (RHA)	512.79	0.399	This work
Zeolite NaY	Rice husk ash (RHA)	39.86	0.163	68
Zeolite A	Rice husk ash (RHA)	3.62	0.00681	69
Zeolite X	Rice husk ash (RHA)	391.4	0.19	70
Zeolite NA-ZSM-5	Rice husk ash (RHA)	377.20	0.219	71
Zeolite CAN	Rice husk ash (RHA)	59.909	0.101	72
Zeolite ZSM-5	Rice husk ash (RHA)	383.503	0.244	35
Zeolite H-ZSM-5	Rice husk ash (RHA)	320.69	0.17	73
Zeolite A	Fly ash	58.29	0.071	50
Zeolite X	Fly ash	164.34	0.054	50
Zeolite A	Fly ash	39	0.016	62
Zeolite A	Industrial metasilicate	10.8	0.0026	51
Zeolite A	Sodium silicate (chemical)	5.16	0.006	74
Zeolite X	Silica gel	285.2	0.13	75
Zeolite X	Commercial zeolite	268.98	0.2101	76

#### 3.2.6 CO_2_ sorption studies

**Fig. 21:**
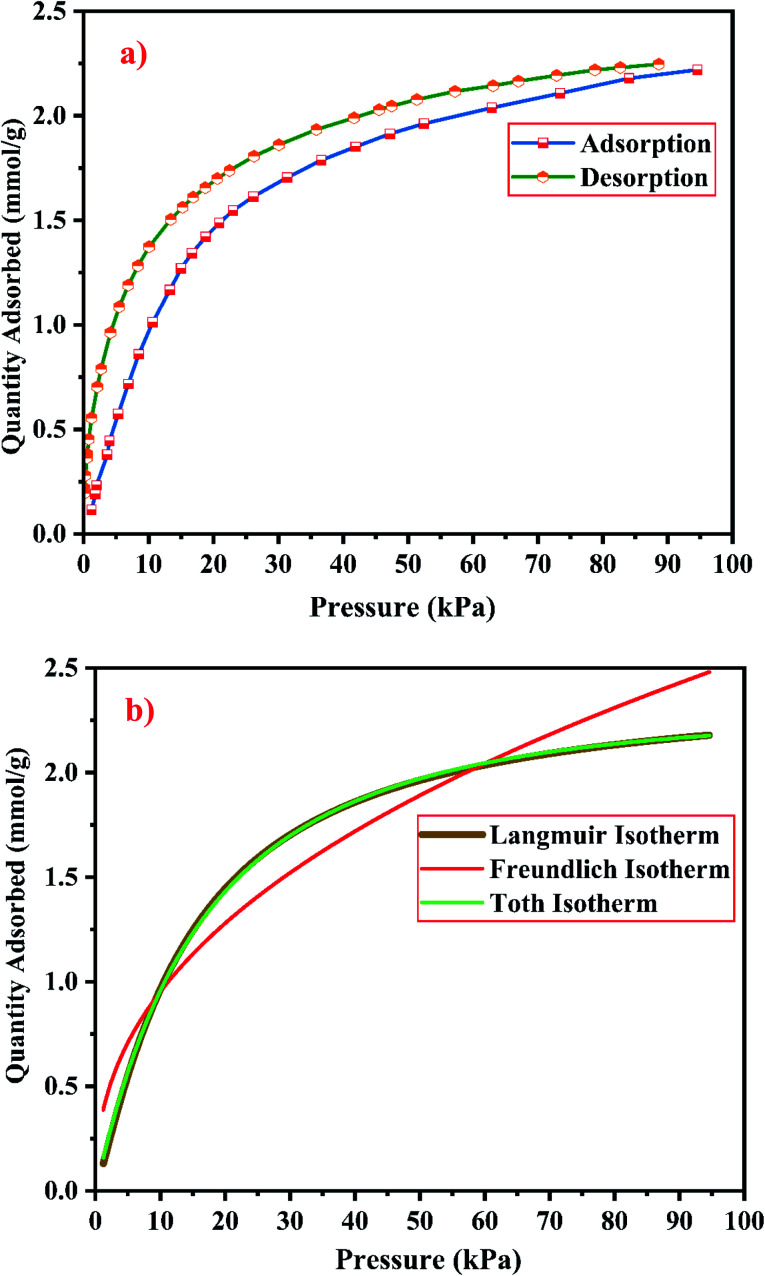
(a) CO_2_ adsorption/desorption studies and (b) fitted adsorption isotherm studies of zeolite RA synthesized from RHA.

**Fig. 22:**
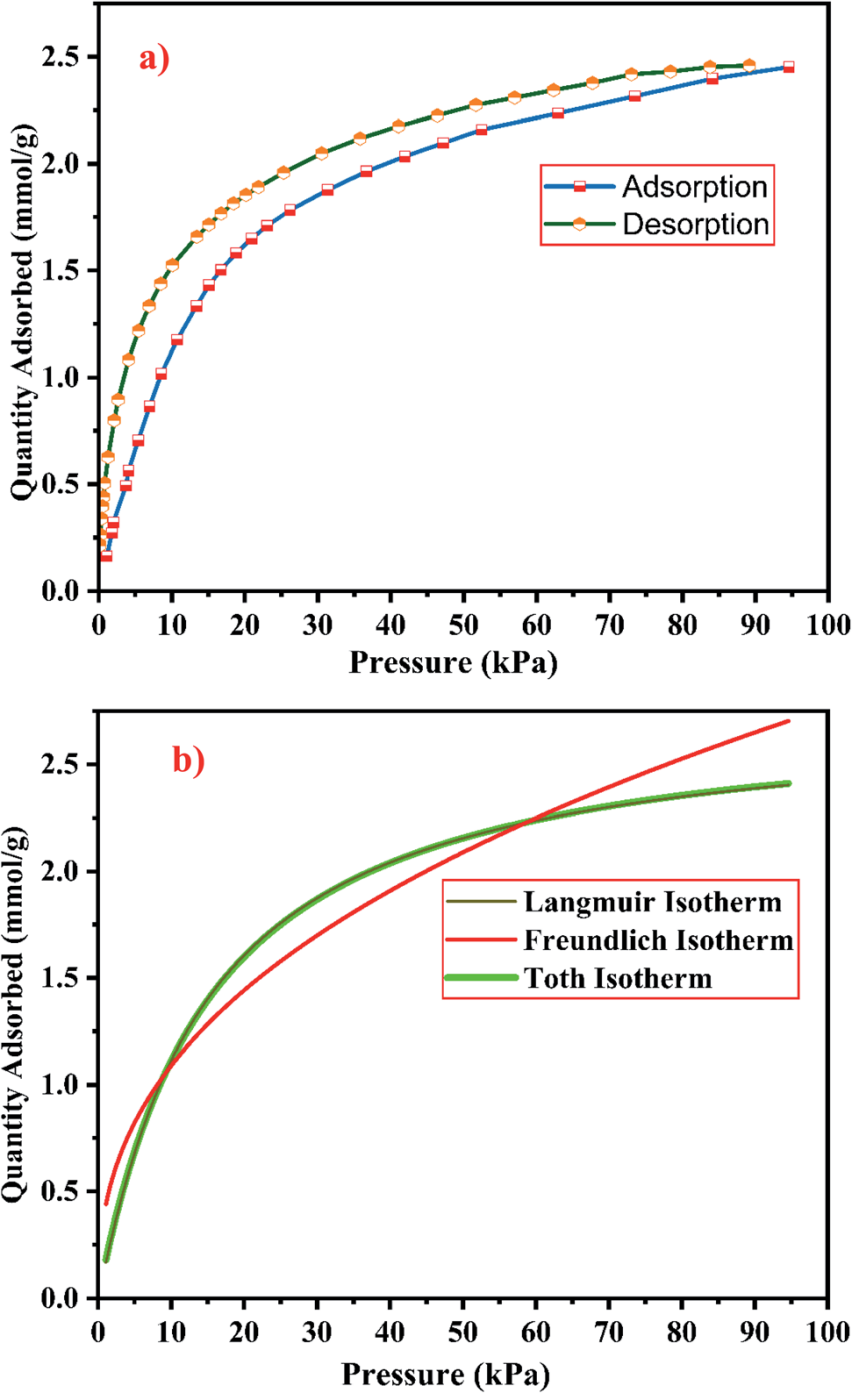
(a) CO_2_ adsorption/desorption studies and (b) fitted adsorption isotherm studies of zeolite RX synthesized from RHA.

The gas–solid interaction strength and the number of existing adsorption pore sites, influence the gas adsorption capacity of the material. The gas–solid interaction strength of the material is studied by using the characteristics of the adsorbent's surface chemistry and pore structure, and by using the other adsorbate's properties such as molecule size, polarizability, and quadruple moments. CO_2_ has a polarizability of 26.5 × 10^−25^ cm^3^ and a quadruple moment of 4.3 × 10^−26^ esu cm^2^.^9^Fig. 21(a) and 22(a) show the experimental CO_2_ sorption studies of the synthesized zeolite RA and RX at 297.15 K in the pressure range between 0 to 101 kPa.

From the Fig. 21(a) and 22(a), it is observed that for both zeolite RA and RX the sorption curves are in accordance with type I adsorption isotherm according to the IUPAC classification. The maximum adsorption recorded are 2.22 and 2.45 mmol g^−1^ for the synthesized zeolite RA and RX respectively.^20,25^ The sorption isotherm has a sharp gradient due to its high adsorption in the low-pressure range and the gradient decreases gradually in the high-pressure range and the curve attains a flat region at the saturation point of the material. The primary interactions of the zeolite and CO_2_ molecules are mostly due to the electrostatic, dispersion, induction, and short-range repulsive force between them. The zeolite adsorbs CO_2_ molecules because of the strong quadrupolar interaction between adsorbate molecules and permanent electric field induced by the charge balancing cation of zeolites. The nature of cations (Na^+^) and their position in the zeolite pore indicate the permanent electric field of the material. From the isotherm, it is observed that there is a sudden increase in the CO_2_ adsorption at low relative pressure and this is mainly due to the strong electrostatic interaction of CO_2_ quadruples with unoccupied cations present in the zeolite framework structure. At high relative pressure, there is no availability of unoccupied cations, hence the curve reaches the saturation point and reaches a plateau nature correspondingly.^9,60^

In the framework structure of the zeolite, the Si and Al atoms are present at the centre of a tetrahedral structure and they are not directly interacting with the CO_2_ molecules and even if they interact their interactions are even negligible in most cases. As a result, the adsorbate interacts mostly with accessible additional framework cations (*via* one oxygen end of the CO_2_ molecule) and the oxygen atom of the zeolite framework (through the carbon atom).^9^ ESI Fig. S3† shows the reaction mechanism of the CO_2_ molecule with the zeolite framework structure. The reaction mainly occurs due to the ion–dipole interaction occurring between the adsorbate and the cations present in the zeolite framework structure. Hence, the presence of alkali cation (Na^+^) in the synthesized zeolite framework structure is responsible for the adsorption of adsorbate molecules. Therefore, the maximum CO_2_ adsorption is obtained for zeolite RX compared to the zeolite RA which indicates that more alkali cations (Na^+^) of RX interacts with the adsorbate gas. Subsequently, the presence of more cations (Na^+^) in the framework of zeolite results in an increase of the basicity of the material, and hence adsorbs more acidic CO_2_ adsorbate molecules and stores it inside the zeolite pores.^49^

The framework structure of zeolite RA, is formed by the repeated unit of sodalite cages interconnected by double 4 rings (D4R) and has a kinetic diameter of 0.4 nm which is almost similar to the CO_2_ molecule kinetic diameter of 0.33 nm. Similarly, zeolite RX structure is formed with 12 oxygen membered rings (D6R) with uniform aperture size.^41,61^ It is clear from the Fig. 21(a) and 22(a) that, the zeolite RX shows higher adsorption compared to zeolite RA. The high intake of CO_2_ molecules by zeolite RX may be attributed to the desirable Si/Al ratio and high surface area of zeolite RX when compared to zeolite RA. The zeolite RX has a Si/Al ratio of around 1.27 and the aluminium atom present in the framework structure creates a negative charge and they are balanced by the alkali cation (Na^+^) present in the zeolite framework which causes the high adsorption of CO_2_ molecules.^9,25^

**Fig. 23:**
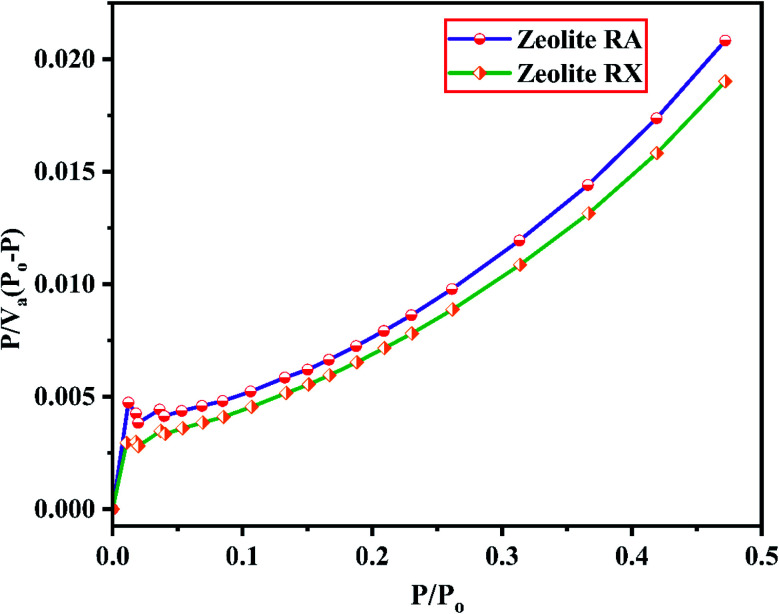
BET plots of zeolite RA and RX synthesized from RHA.

The BET plots for the synthesized zeolite RA and RX are given in Fig. 23. Using the BET theory, one can determine the monolayer volume of adsorbed gas and consequently the specific surface area by considering several hypotheses.^25^ The CO_2_ adsorption textural properties of the synthesized zeolites are given in the Table 5 and the textural property values obtained are found to be higher when compared to the values for the zeolite synthesized *via* hydrothermal method and reported by Czuma *et al.*^62^ The observed experimental adsorption isotherm data are fitted by non-linear curve fit using Langmuir, Freundlich and Toth isotherm models. The expressions used for the calculation of isotherms are mentioned in the ESI.† The fitted adsorption isotherm models are shown in Fig. 21(b) and 22(b) and their corresponding parameter values are given in Table 6. The fitted isotherm models are found to be of type I adsorption isotherm according to the IUPAC classification criterion. For all isotherm models, the correlation coefficient is found to be nearly 1 and it indicates the larger adsorption of CO_2_ molecules which indicates that the system exhibits more heterogeneity.^25^ From the calculated parameter values the Langmuir isotherm is found to have a higher correlation coefficient compared to the other isotherm models which indicate the best adsorption fit with experimental data.^41^ A comparison of experimentally obtained results of CO_2_ adsorption corresponding to the present study with those of other researchers are presented in Table 7.

**Table 5:** CO_2_ textural properties of zeolite RA and RX synthesized from RHA

Sample	*S*_BET_ (m^2^ g^−1^)	Monolayer volume *V*_m_ (cm^3^ (STP) per g)
Zeolite RA	256.57	44.212
Zeolite RX	212.5	36.616

**Table 6:** CO_2_ adsorption isotherm properties of zeolite RA and RX synthesized from RHA

Samples	Correlation coefficients (*R*^2^)		
	Langmuir adsorption	Freundlich isotherm	Toth isotherm
Zeolite RA	0.99912	0.93725	0.99806
Zeolite RX	0.99903	0.94507	0.99883

**Table 7:** Comparison of results of experimental CO_2_ adsorption of zeolite RA and RX synthesized from RHA

Sample	Silicon source	Temperature (K)	CO_2_ uptake (mmol g^−1^)	References
Zeolite RA	Rice husk ash (RHA)	297.15	2.22	This work
Zeolite RX	Rice husk ash (RHA)	297.15	2.45	This work
Zeolite ZSM-5-PEI-30	Rice husk ash (RHA)	363.15	1.37	35
Zeolite NaA	Rice husk ash (RHA)	273.15	1.46	41
Zeolite NaX	Rice husk ash (RHA)	273.15	3.12	41
Zeolite NaZSM-5	Rice husk ash (RHA)	273.15	2.20	41
Geopolymers	Rice husk ash (RHA)	308.15	0.80	77
Zeolite (Serbia)	Commercial zeolite	298.15	1.17	78
Zeolite chitosan	Commercial zeolite	298.15	1.7	78
Silicate	Tetraethylorthosilicate (TEOS)	308.15	1.3	79
Fly ash zeolite	Fly ash	323	1.20	62
Graphene/ZIF-8 aerogel	—	298	0.99	80
Microporous carbon spheres	—	303.15	1.2	81
Polyethylenimine-functionalized SBA-15	—	298	0.81	82
Polyimide/ZIF-67	—	298	0.446	83

**Fig. 24:**
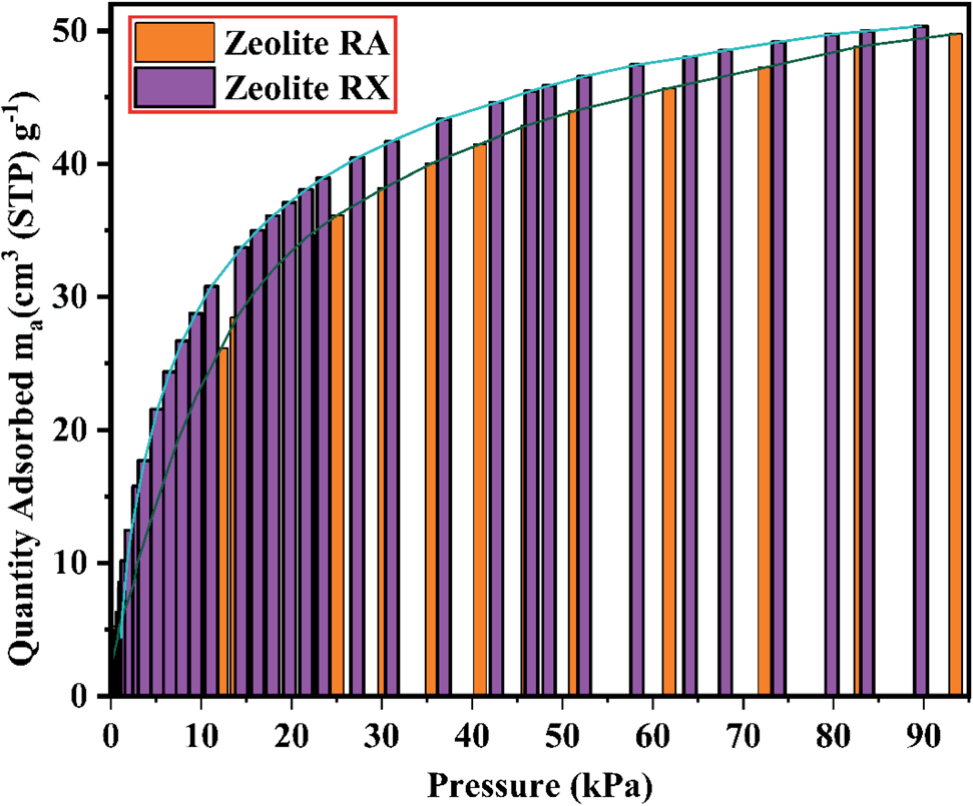
Comparison data of CO_2_ adsorption of zeolite RA and RX synthesized from RHA.

Based on the results obtained, it is observed that various factors such as the size, Si/Al ratio, shape of pores, the polarity of the adsorbed molecule and the other empirical properties such as pressure and temperature are responsible for the adsorption capacity of zeolites. It is also observed that at lower relative pressure the adsorption of adsorbate gas is more compared to higher relative pressure.^63^ This is because adsorbed molecules have a greater inclination to occupy areas having less adsorbate–adsorbate interactions, such as smaller pores, rather than adsorbate pore interactions at low pressures. At high relative pressure, the CO_2_ adsorption is restricted by the zeolite pore size which results in the prevention of further CO_2_–CO_2_ interaction in the active pore sites of zeolites.^9^ From the synthesized zeolite it is observed they have a lower Si/Al ratio. The lower Si/Al ratio zeolite exhibits a larger electric gradient, and this results in strong adsorption of CO_2_ molecules into it. The synthesized zeolite RX has Si/Al ratio of 1.27 and it is more basic (based on pH) in nature in their framework structure compared to zeolite RA which results in higher adsorption of CO_2_ molecules.^25^ Meanwhile, from the Fig. 24 it could be concluded that the maximum adsorption of CO_2_ is recorded for the zeolite RX compared to zeolite RA. The maximum adsorption values are 49.76 and 54.95 m_a_ (cm^3^ (STP) per g) for zeolite RA and RX respectively.

It is observed that the synthesized zeolite samples RA and RX have pores lying in the micro to mesoporous region, with total surface area of 106.25 and 512.79 m^2^ g^−1^ (determined by the BET method) respectively. During interaction the formation of linear OCO–X^+^ complexes (where X^+^ = Na^+^) take place, which is also involved in the perturbation of Si–O–Al bonds.^64^ As reported by Coluccia S. *et al.*^65^ zeolitic supercages made the molecules to linearly coordinate with X^+^ cations (Lewis acidity), and also numerous types of carbonate-like species interact in intricate ways which has also been reported by several other researchers like Martra, G. *et al.*^66^ and Montanari, T. and Busca G.^67^

## 4. Conclusion

In this paper we have reported about the synthesis of zeolite RA and RX, prepared using hydrothermal method and the silicon source material used for the synthesis has been extracted from rice husk ash. The prepared RHA samples and the silicon source extracted from RHA are observed to be amorphous in nature. The FESEM micrographs revealed the presence of “wave-like” morphology and “flakes” like structure for the RHA and SS. The elemental composition results indicate that the samples are of pure nature. The synthesized zeolite RA and RX samples, are found to exhibit good crystallinity. In the FTIR spectra, the bands present at 522 cm^−1^ and 554 cm^−1^ for the synthesized zeolite RA and RX indicate the presence of D4R and D6R units composed of double four- and six-membered rings of tetrahedra respectively. The thermal properties of the synthesized samples show that the maximum weight loss recorded are 15% and 5% for the prepared zeolite RA and RX. The zeolite RX has a high Si/Al ratio compared to the zeolite RA and hence zeolite RX exhibits high thermal stability. From, the FESEM and HRTEM micrographs, it is found that the RA and RX samples exhibit “Ice cube” and “octahedral” morphology. From the N_2_ adsorption–desorption studies, it is found that typical type IV and type I adsorption isotherms correspond to zeolite RA and RX respectively. The BET surface areas of the prepared samples are found to be 106.25 m^2^ g^−1^ and 512.79 m^2^ g^−1^. By using the BJH equation the average pore sizes have been calculated and are found to be 32 and 38 nm for the zeolite RA and RX respectively.

The results of experimental CO_2_ sorption studies for zeolite RA and RX are found to be in accordance with type I adsorption according to the IUPAC classification criterion. The maximum experimental CO_2_ adsorption are found to be 2.22 and 2.45 mmol g^−1^ for the synthesized zeolite RA and RX respectively. The obtained isotherms are fitted using Langmuir, Freundlich and Toth non-linear adsorption isotherms. From the fitted isotherm models, it is found that the correlation coefficient is nearly 1, which indicates the heterogeneous nature of the materials. So, the method used in the present study for the synthesis of zeolite RA and RX using silicon source material extracted from rice husk is considered to be a low-cost production method and also an environmentally friendly method and the significant advantage is that it can be easily scaled for large scale production.

## Data availability

All data generated or analysed during this study are included in this published article (and its ESI†).

## Author contributions

J. M. carried out the experimental work related to the preparation of samples. J. M took part in the preparation of the manuscript and carried out the characterization part. A. S., M. N & D. V. supervised, helped in evolving the methodology for the work and were involved in the proof correction of the manuscript.

## Conflicts of interest

The authors declare no competing interests.

## Supplementary Material

RA-012-D2RA04052B-s001
