# Supplementary material for: CO2 adsorption performance of template free zeolite A and X synthesized from rice husk ash as silicon source
Source: RSC Adv. 2022 Aug 17;12(36):23221–39. doi: 10.1039/d2ra04052b (PMC9384810; doi:10.1039/d2ra04052b)
Supplement: RA-012-D2RA04052B-s001 [file RA-012-D2RA04052B-s001.pdf]

Electronic Supplementary Material (ESI) for RSC Advances.
This journal is © The Royal Society of Chemistry 2022

CO_2 adsorption performance of template free zeolite A and X synthesized from Rice husk ash as silicon source

Jayaprakash Madhu[a], Agilan Santhanam[a], Muthukumarasamy Natarajan[a], Dhayalan Velauthapillai[b*]

[a]*Department of Physics, Coimbatore Institute of Technology, Coimbatore, Tamil Nadu, India.*

[b]*Faculty of Engineering and Science, Western Norway University of Applied Sciences, 5063, Bergen, Norway*

*Corresponding author E-mail: Dhayalan.Velauthapillai@hvl.no

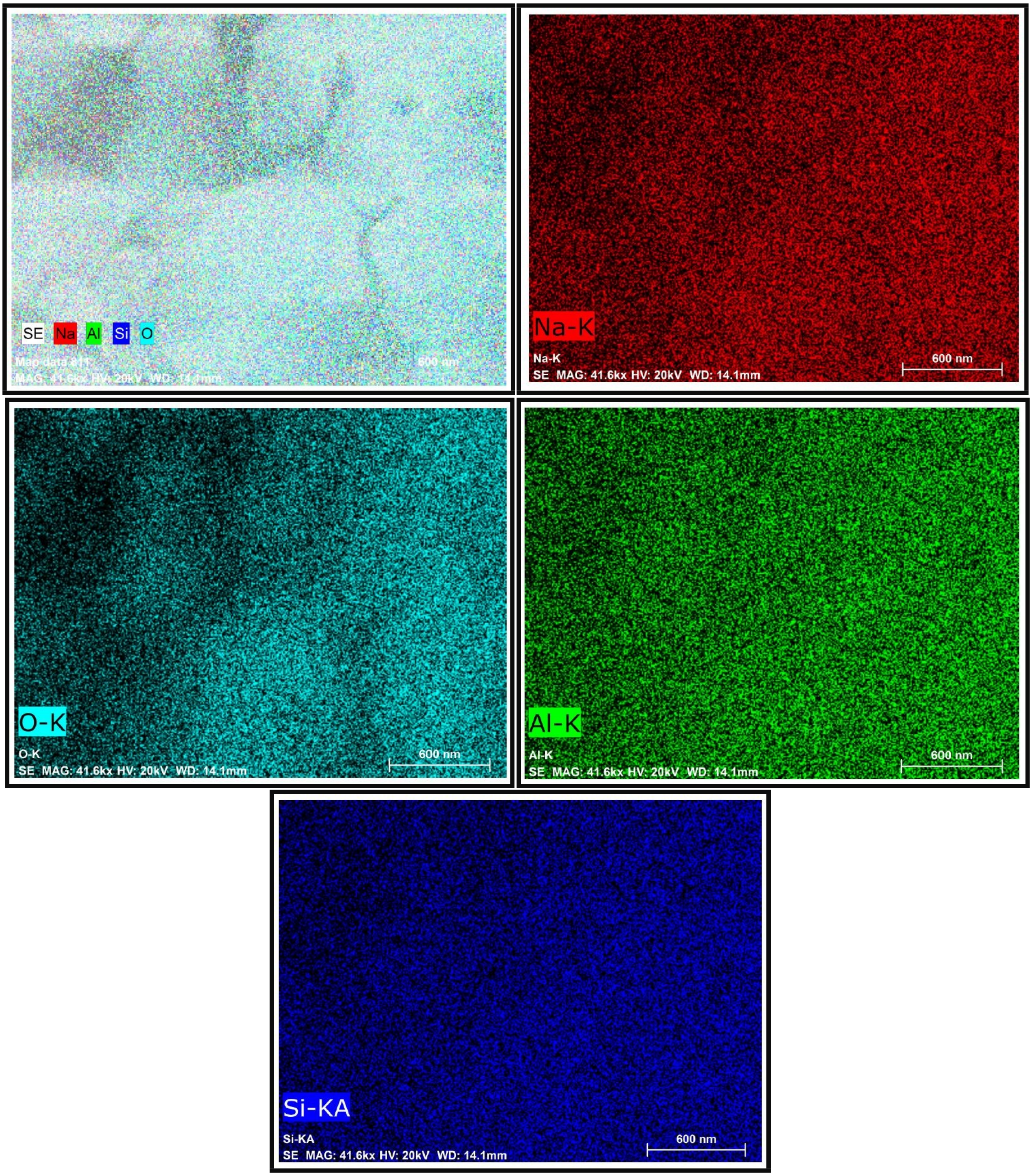
SE Na Al Si O
600 nm
Na-K
Na-K
SE MAG: 41.6kx HV: 20kV WD: 14.1mm
600 nm
O-K
O-K
SE MAG: 41.6kx HV: 20kV WD: 14.1mm
600 nm
Al-K
Al-K
SE MAG: 41.6kx HV: 20kV WD: 14.1mm
600 nm
Si-KA
Si-KA
SE MAG: 41.6kx HV: 20kV WD: 14.1mm
600 nm

Figure S1: Elemental mapping of synthesized zeolite RA from RHA

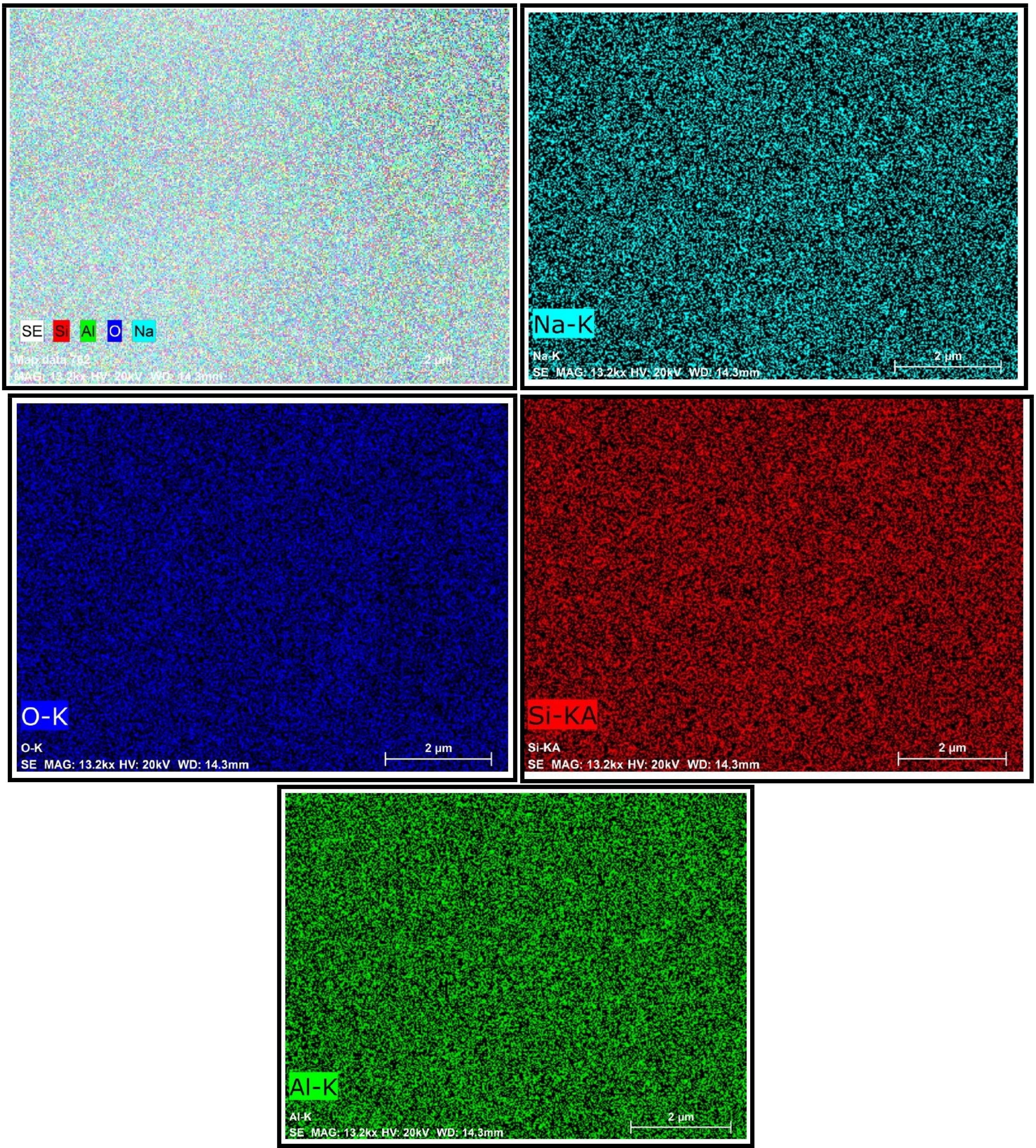

Figure S2: Elemental mapping of synthesized zeolite RA from RHA

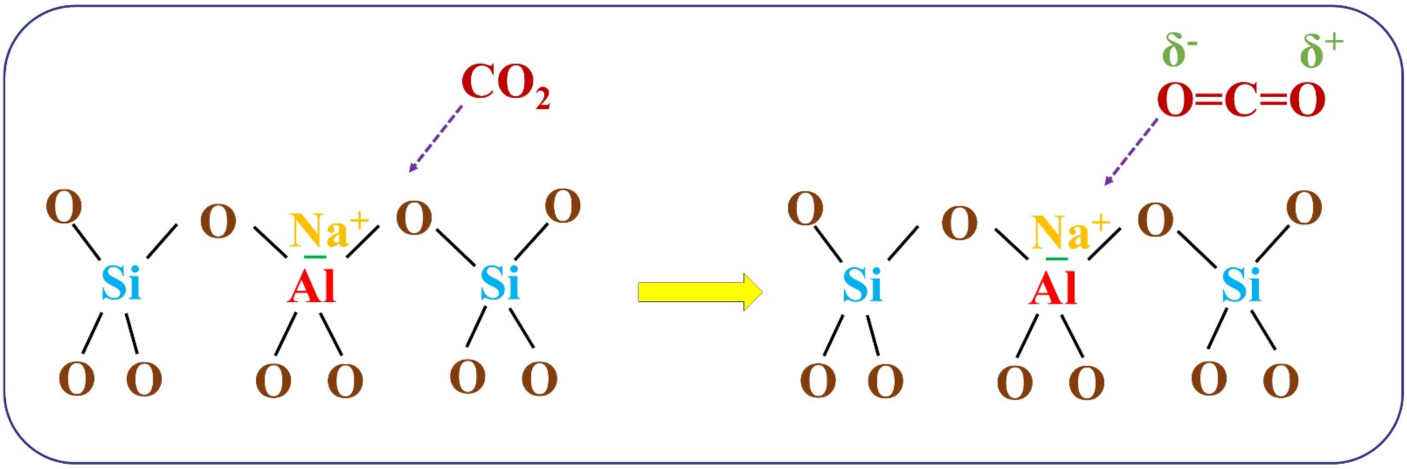

Figure S3: General mechanism of CO_2 interaction with zeolite

Adsorption isotherm equations:

The CO_2 adsorption results obtained for the synthesized zeolites are subjected to study under the non-linear fitting using Langmuir, Freundlich and Toth adsorption isotherm curve fitting with the aid of origin software 2021® to study the CO_2 adsorption behaviour.

The Langmuir isotherm model is primarily developed for the adsorption of gases into solids. Moreover, the adsorption isotherm is taken place in a monolayer or the adsorption may only occur in a fixed number of localized sites of the adsorbent surface with all adsorption sites are identical and energetically equivalent. Hence, the Langmuir equation is based on the prospects of a structurally homogenous adsorbent. The isotherm equation can be represented in the following equation.

$$q = q_m \frac{b_p}{1 + bp} \qquad \text{---------------(1)}$$

where q (mmolg^{-1}) is the adsorbed amount in equilibrium with the gas phase, q_m (mmolg^{-1}) is the maximum adsorbed amount, p is the equilibrium pressure of the gas phase, and b is the Langmuir isotherm constant.

The Freundlich model is one of the primitive known equations describing the adsorption process. It is an empirical equation used for the non-ideal adsorption that involves heterogeneous sorption. The non-linear form of the Freundlich isotherm equation is represented as follows,

$$q = kp^{\frac{1}{n}} \quad \text{---------------(2)}$$

where q (mmolg^{-1}) is the adsorbed amount, K and n are constants for a given adsorbate and adsorbent at a particular temperature, p (bar) is the equilibrium pressure of the gas phase, and n represents the heterogeneity factor, the value becomes more heterogeneous as its value gets further from one.

The Toth isotherm model is a three-parameter equation derived from the Langmuir equation. This model adopts a quasi-Gaussian energy distribution and is an effective isotherm model representing the adsorption of gases at both low and high pressure on a heterogenous surface. This model was chosen because it incorporates the energy heterogeneity of the sorbent's active sites and is represented by Equation.

$$q = q_m \frac{bp}{(1 + bp)^n)^{1/n}} \quad \text{---------------(3)}$$

where q_m (mmolg^{-1}) is the maximum adsorbed amount, b is the Toth constant (bar^{-1}), p is the pressure (bar), and n represents the heterogeneity factor. When the value **n=1**, the expression reduces to an isotherm model relation.
